# Molecular Mechanisms of Acclimatization to Phosphorus Starvation and Recovery Underlying Full-Length Transcriptome Profiling in Barley (*Hordeum vulgare* L.)

**DOI:** 10.3389/fpls.2018.00500

**Published:** 2018-04-18

**Authors:** Panrong Ren, Yaxiong Meng, Baochun Li, Xiaole Ma, Erjing Si, Yong Lai, Juncheng Wang, Lirong Yao, Ke Yang, Xunwu Shang, Huajun Wang

**Affiliations:** ^1^Gansu Provincial Key Lab of Aridland Crop Science/Gansu Key Lab of Crop Improvement and Germplasm Enhancement, Gansu Agricultural University, Lanzhou, China; ^2^College of Agronomy, Gansu Agricultural University, Lanzhou, China; ^3^College of Life Sciences and Technology, Gansu Agricultural University, Lanzhou, China; ^4^Department of Agriculture and Forestry, College of Agriculture and Animal Husbandry, Qinghai University, Xining, China

**Keywords:** phosphorus stress and restoration, response systems, time and space, single-molecule real-time sequencing, barley

## Abstract

A lack of phosphorus (P) in plants can severely constrain growth and development. Barley, one of the earliest domesticated crops, is extensively planted in poor soil around the world. To date, the molecular mechanisms of enduring low phosphorus, at the transcriptional level, in barley are still unclear. In the present study, two different barley genotypes (GN121 and GN42)—with contrasting phosphorus efficiency—were used to reveal adaptations to low phosphorus stress, at three time points, at the morphological, physiological, biochemical, and transcriptome level. GN121 growth was less affected by phosphorus starvation and recovery than that of GN42. The biomass and inorganic phosphorus concentration of GN121 and GN42 declined under the low phosphorus-induced stress and increased after recovery with normal phosphorus. However, the range of these parameters was higher in GN42 than in GN121. Subsequently, a more complete genome annotation was obtained by correcting with the data sequenced on Illumina HiSeq X 10 and PacBio RSII SMRT platform. A total of 6,182 and 5,270 differentially expressed genes (DEGs) were identified in GN121 and GN42, respectively. The majority of these DEGs were involved in phosphorus metabolism such as phospholipid degradation, hydrolysis of phosphoric enzymes, sucrose synthesis, phosphorylation/dephosphorylation and post-transcriptional regulation; expression of these genes was significantly different between GN121 and GN42. Specifically, six and seven DEGs were annotated as phosphorus transporters in roots and leaves, respectively. Furthermore, a putative model was constructed relying on key metabolic pathways related to phosphorus to illustrate the higher phosphorus efficiency of GN121 compared to GN42 under low phosphorus conditions. Results from this study provide a multi-transcriptome database and candidate genes for further study on phosphorus use efficiency (PUE).

## Introduction

Phosphorus (P), a necessary macronutrient, is required for growth and development of all living organism. P is highly reactive with other elements, for example, hydrogen, and oxygen, and can therefore not exist alone (Jez et al., 2016). Furthermore, phosphate rock is the only source of phosphorus, and will be depleted in 50–100 years worldwide (Kochian, 2012). The bioavailable form of phosphorus that plants can directly absorb is orthophosphate, which is a nonrenewable resource and derived from phosphate rock mining. Plants can only utilize ≤30% of inorganic phosphorus (Pi) fertilizers due to the inaccessible form developed from its reactivity with aluminum and iron oxides and transformation of soil microbes (Jez et al., 2016). However, the remaining phosphorus in soil is fixed by roots and soil particles or transported to lakes and rivers through farm drainage and surface runoff, and leads to severe environment pollution such as eutrophication of water (Andersson et al., 2013). Pi-fertilizer is over-applied in order to make crops more productive due to the growing world population (Wu et al., 2013). According to the situation mentioned above, breeding of new crop varieties with high phosphorus acquisition and use efficiency should be implemented, which will not only reduce the high cost of Pi-fertilizers, but also render the agriculture development sustainable (Liu et al., 2016).

For acclimatization to external environments with low levels of phosphorus, plants have developed a series of response mechanisms of phenotypic, physiological, biochemical, metabolic, and molecular alternations to absorb more Pi from soil and reallocate the phosphorus in plants (Zhang et al., 2014). In general, plants often reduce the growth of shoots and roots under Pi deprivation, thus resulting in an increase of the root/shoot ratio (Royo et al., 2015). At the physiological and biochemical levels, Pi content is a very important parameter, and also decreases under conditions of phosphorus stress (Wang H. et al., 2014). Meanwhile, plant roots secrete multiple organic acids such as citrate and malate into rizospheres for exchanging via ligand exchange reactions the phosphate that is adsorbed onto oxides surfaces (Clarholm et al., 2015; Ding et al., 2016). Moreover, phospholipids located in the membrane are degraded to available phosphorus and replaced by galactolipids and sulfolipids, large amounts of which are synthesized under phosphorus deprivation (Okazaki et al., 2013). In addition, many metabolic processes (e.g., photosynthesis, respiration, amino acids, and lipid metabolism) were suppressed and molecules containing phosphorus (e.g., nucleases, ribonucleases, phosphoesterases, and purple acid phosphatases) were degraded under Pi-limiting conditions, meanwhile, genes involved in all of which and Pi transport were differetially expressed between normal ans low phosphorus treatment.

Every species exhibits an individual pattern of responding to phosphorus starvation stress that is linked to the growth strategy. These responses, under the condition of low phosphorus, depend on tissues, and also on genotypes (Byrne et al., 2010); the selection of varieties with high phosphorus efficiency is a feasible method for improving plant PUE. Previous studies have reported that monocotyledons are more P-efficient than dicotyledons (Vergutz et al., 2012). C4 plants are more sensitive to low phosphorus than C3 plants, and the photosynthesis rate of C4 plants decreases sharply under low phosphorus conditions (Sage and Mckown, 2005). Barley, one of the earliest domesticated crops (Pourkheirandish et al., 2015) and the world's fourth most ample cereal crop (Consortium, 2012), is widely used as fodder for animals and raw materials for the beer industry (Mascher et al., 2017). Barley is also greatly accommodated and is a staple plant, even known as “the last crop before the desert,” in some territories with a volatile climate. In addition, barley is now cultivated all over the world and its ability to increase yields in harsh circumstances is critical for future decades (Keller and Krattinger, 2017). Previous studies have demonstrated that barley has unique adaptive mechanisms in response to abiotic stress (Zeng et al., 2014; Quan et al., 2016). In previous experiments, we have identified two barley genotypes with contrasting phosphorus efficiency, which provide excellent germplasms for subsequent molecular research (Ren et al., 2016).

Phosphorus transporters are major proteins for uptake and translocation of phosphorus, and were firstly identified in *Arabidopsis thaliana* and *Solanum tuberosum* treated with a yeast mutant without the *pho84* gene (Muchhal et al., 1996; Leggewie et al., 1997). In *Arabidopsis*, the majority of PHT1 genes were expressed in different parts of roots (Abel, 2017). Shin et al. (2004) found that both *Pht1;1* and *Pht1;4* can improve the uptake of phosphorus in *Arabidopsis* (Shin et al., 2004). In addition, two other genes of the PHT1 family, *AtPHT1;8* and *AtPHT1;9*, are involved in transporting phosphorus from roots to shoots (Lapis-Gaza et al., 2014). In a recent study, a SULTR-like phosphorus distribution transporter (SPDT) was reported to distribute phosphorus to the grain (Yamaji et al., 2017). In terms of regulation of phosphorus genes, WRKY45 was reported to activate the expression of *PHT1;1* in *Arabidopsis* (Wang H. et al., 2014). Moreover, miR399 and miR827 can also positively regulate phosphorus genes (Chien et al., 2017). In barley, eight pht1 homologous genes have been identified (Smith et al., 1999; Rae et al., 2003). Among these PHT1 genes, *HvPHT1;6* was identified to redistribute the phosphorus for expression in shoots and roots (Preuss et al., 2010), and *HvPHT1;8* was detected as a mycorrhiza-specific gene (Glassop et al., 2005). In addition, the promoters of *HvPht1;1* and *HvPht1;2* were highly expressed under phosphorus starvation conditions (Nussaume et al., 2011). Schünmann et al. (2004) also reported that the expression of phosphorus transporters was regulated by a MYB-type transcription factor (Schünmann et al., 2004). In terms of phosphorus efficiency, previous researches mainly focus on one of three major phosphorus metabolic processes (Pi uptake, transport and utilization), while interaction and transcriptional regulation among them still need to be further investigated.

Scenic changes clearly occur in growth and development processes under Pi-limiting conditions, as well as in different omics profiles. Next-generation sequencing (NGS) technology (RNA-seq) can generate digital data of gene expression with removal of the limits of predesigned probes (Secco et al., 2013), and have been conducted not only on *Arabidopsis* (Lan et al., 2012), rice (Oono et al., 2013), maize (Li et al., 2012), but also on *de novo* assembly for many organisms in the past (Du et al., 2017). The results of incomplete sequences and low quality transcripts gained from RNA-seq are a limitation of alternative splice analysis and corrected annotations, respectively (Tilgner et al., 2015). However, the advent of single-molecule real-time (SMRT) sequencing—developed by Pacbio Biosciences—brought an end to these restrictions by generating longer or full-length sequences, and by improving the annotation information of the known genome (VanBuren et al., 2015; Lan et al., 2017). However, SMRT sequencing can provide inaccurate information of genes, an important issue that was reported to account for the high error rate in recent studies. Nevertheless, the problem can be corrected by RNA-seq reads and circular-consensus (CCS) reads. In recent years, the method of combining RNA-seq and SMRT sequencing has been frequently applied to generate comprehensive information at the transcriptional level, which provides the scientific basis for perfecting the genome database and molecular breeding (Xu et al., 2015).

In the present study, SMRT sequencing technology was carried out in the two barley varieties with varying phosphorus efficiency under different treatments, at three time points, for analyzing sophisticated responses of barley plants to Pi starvation and recovery. A rich and integrated data-set was constituted for improving genome annotation and extending our awareness of molecular responses. This research was achieved by means of a previously unused method in barley, unveiling important transcripts responding to external Pi stress, and the results can be exploited for breeding new crop varieties with high PUE.

## Materials and methods

### Plant materials and growth conditions

Two barley genotypes (GN121, low-P-tolerant, and GN42, low-P-sensitive) were screened and used for this research (Ren et al., 2016). Hydroponic experiments were conducted from early January to late May in 2017 at Gansu Agricultural University (Lanzhou, China). The climate chamber was set at 20 ± 5°C, 50~70% relative humidity and a photon fluence rate of 300 μmol photonsm^−2^ s^−1^ with a 16 h day/8 h night cycle. The Pi-plenty nutrient fluid (CK: normal Pi/R: Pi resupply) contained 0.39 mM KH_2_PO_4_, and the Pi-deprived nutrient fluid (–Pi) included 0.039 mM KH_2_PO_4_ and 0.18 mM K_2_SO_4_. The ingredients of the nutrient solution [Hoagland (Hoagland and Arnon, 1950), modified] was as follows: macronutrients: 1 mM KNO_3_, 5 mM Ca(NO_3_)_2^.^_4H_2_O, 5 mM CaCl_2_, 1 mM MgSO_4^.^_7H_2_O; micronutrients: 50 μM H_3_BO_3_, 50 μM MnSO_4^.^_H_2_O, 0.05 μM CuSO_4^.^_5H_2_O, 15 μM ZnSO_4^.^_7H_2_O, 3 μM Na_2_MoO_4^.^_2H_2_O, 50 μM Na_2_-EDTA, 50 μM FeSO_4_. The pH was adjusted to 6.0 ± 0.1 with NaOH or HCl. Seeds were pre-sterilized and then germinated in transparent plastic boxes placed in a phytotron (22/18°C, day/night). After 5 d, uniform seedlings without endosperms of each accession were selected and transplanted into 20-L containers with hydroponic solution containing different phosphate concentrations (CK: 0.39 mM, –Pi: 0.039 mM) for 19 d. Half of the seedlings treated with low phosphorus were then resupplied with 0.39 mM Pi for 3 d, while the other half of the seedlings continuously remained in –Pi conditions to act as controls. Shoots and roots were separately collected with three replicates at three time points: day 3 and day 19 after transplanting to CK and –Pi conditions, as well as the day 3 after Pi recovery. In addition, all samples were gathered at 2 pm for reducing experimental error, and immediately frozen in liquid nitrogen for molecular analysis.

### Determining biomass and Pi concentration

All tissues (roots and shoots) were dried at 80°C for 72 h until their weight remained constant, in order to estimate the dry weight, and were then crushed and digested for measuring Pi concentration (Murphy and Riley, 1962).

### Extraction and detection of total RNA

A total of 72 samples [2 genotypes (GN121 and GN42) × 2 tissues (leaf and root) × 2 treatments (–Pi and CK/R) × 3 time points (3 d, 19 d and 22 d) × 3 biological replications] were used for transcriptome analysis. Total RNA was extracted using TRIzol reagent (Invitrogen, 15596026) according to the manufacturer's instructions (Wang et al., 2016). A NanoPhotometer® spectrophotometer (IMPLEN, CA, USA) was used for checking RNA purity. RNA concentration was measured on a Qubit® 2.0 Fluorimeter (Life Technologies, CA, USA). Assessment of RNA integrity was performed by the Bioanalyzer 2,100 system (Agilent Technologies, CA, USA). Based on the above-mentioned parameters, RNA was selected for subsequent studies at a A260–A280 ratio value of more than 1.9.

### Illumina RNA-seq library construction

RNA of each sample (3 μg) was prepared for building the next-generation sequencing library, which was conducted on the NEBNext® Ultra™ RNA Library Prep Kit for Illumina® (NEB, USA) according to the manufacturer's instructions. The detailed steps are as follows: Poly-T oligo-attached magnetic beads were utilized to purify mRNA from total RNA, fragmentations of which were obtained using divalent cations with NEBNext First Strand Synthesis Reaction Buffer (5×). Random hexamer primers and M-MuLV Reverse Transcriptase were employed to synthesize first strand cDNA, and both DNA Polymerase I and RNase H were applied to synthesize the second strand cDNA. Exonucleases/polymerases were used for forming blunt ends transformed from remaining overhangs. Hybridization was performed for the NEBNext Adaptor, containing a hairpin loop structure after adenylation of 3′ ends of DNA fragments. Purifying the library fragments was carried out using the AMPure XP system (Beckman Coulter, Beverly, USA) for selecting cDNA fragments with a length between 150 and 200 bp. Size-selected and adaptor-ligated cDNAs were treated with USER Enzyme (NEB, USA) before carrying out the polymerase chain reaction (PCR), which was conducted using Phusion High-Fidelity DNA polymerase and primers. Finally, purification of PCR products and evaluation of library quality were performed on AMPure XP system and Agilent Bioanalyzer 2,100 system, respectively (NCBI accession number: SRP131833).

### Barcoding library and single-molecule sequencing

For obtaining the complete information of all transcripts, full length transcriptome sequencing was adopted in the present study. In order to reduce experimental error, the best RNA sample of three replicates was selected from all samples used in Illumina sequencing, and then mixed together in an equal quantity, as one sample, for SMRT sequencing. Optimization of the PCR cycle was conducted before large-scale PCR, the products of which were purified and used for size selection using the BluePippin System. To detect more transcripts with low-abundance, four libraries and 15 SMRT cells (1–2 Kb 5, 2–3 Kb 5, 3–6 Kb 3, and 6-8 Kb 2) were constructed and performed on the PacBio RSII platform (NCBI accession number: SRP131708).

### Quality control

The raw data sequenced on the PacBio RS sequencing instrument was filtered by SMRTLink (4.0) to obtain the Circular Consensus reads, which were divided into two classes (full-length non chimera and non-full-length reads). The method of Iterative Clustering for Error Correction (ICE) was employed for iterative clustering to obtain the consensus reads. Polished consensus reads were acquired from the original consensus reads corrected with non-full-length reads, and used for further studies. In terms of next generation sequencing, internal perl scripts were initially applied to process raw reads with fastq format. In the course of obtaining clean data, three kinds of reads, which were involved in adapter, poly-N and low quality, were deleted from the raw data. Meanwhile, the quality of these clean data was estimated by three parameters including Q20 and Q30. In the present study, the Q20 and Q30 values of the clean data was required to be greater than 95.09 and 88.46%, respectively. Furthermore, the clean data with high quality was screened for subsequent analyses.

### Reads mapping to the reference genome

Barley genome and its annotation documents were used as a reference for analysis of transcriptome data, which were downloaded from the website (http://webblast.ipk-gatersleben.de/barley_ibsc/downloads/). PacBio RSII data was applied to modify the reference genome using Bowtie (v2.2.3) (Langmead and Salzberg, 2012). TopHat (v2.0.12) was selected and used to align the paired-end clean reads to the reference genome because a splice junction database can be formed according to the gene annotation documents by this software (Trapnell et al., 2009). All transcripts were detected by the method of RABT (Reference Annotation Based Transcript) using Cufflinks (v2.1.1) (Trapnell et al., 2012). Asprofile (v1.0) was applied to classify the alternative splicing (AS) events into 12 types, the quantity of which was also calculated (Florea et al., 2013). The analysis of fusion genes and long non-coding RNA was performed with Tofu, CPC and PFAM software according to the instructions (Kong et al., 2007; Punta et al., 2011).

### Differential expression analysis

Reads aligned to every gene were calculated by the software named HTSeq (v0.6.1) (Anders et al., 2015). According to the gene length and reads count aligned to this gene, the FPKM (expected number of Fragments Per Kilobase of transcript sequence per Millions of base pairs sequenced) value of each gene was computed. In the current study, genes with FPKM > 1 were indeed expressed. DESeq R package (v1.18.0) (Anders and Huber, 2012) was employed to analyze differential expression between the treated and control groups with three replicates. On the basis of the negative binomial distribution, a model with statistical routines was performed to detect differential expression using the data of digital gene expression. Expression of DEGs with adjusted *P* < 0.05 corrected by the Benjamini and Hochberg's approach were significant (Benjamini and Hochberg, 1995).

### qRT-PCR

RNA-seq samples were also used for quantitative reverse-transcription PCR (qRT-PCR) to test the dependability of the transcriptome results. Five genes induced by low phosphorus stress were selected from roots and leaves for experimental validation. Specific primers of chosen genes were designed using Primer Premier 5.0 software (Supplemental Table 10) and used for SYBR Green-based qRT-PCR analysis. A Trizol regent (Invitrogen, Cat. No. 15596018) kit was applied to extract total RNA according to the manufacturer's instructions. The first-strand cDNA was obtained using a PrimeScript® RT reagent Kit (TaKaRa, Dalian, China). All qRT-PCR analyses were conducted on a Mastercyclerw ep realplex machine (Eppendorf, Hamburg, Germany) with three replicates. The reactions were incubated at 95°C for 30 s, followed by 40 cycles of 95°C for 5 s, 60°C for 30 s, and ended with Melt-Curve analysis (1 cycle of 95°C for 15 s, 60°C for 30 s, and 95°C for 15 s). The comparative ΔCt method was used to calculate relative expression levels. The endogenous reference gene *HvActin* (GenBank accession number, AY145451) was used to normalize all the results.

### GO and KEGG enrichment analysis of differentially expressed genes

DEGs were also employed for the enrichment analysis of Gene Ontology (GO) using the GOseq R package (Young et al., 2010), which can adjust the gene length bias. The adjusted *P*-value of significantly substantiated GO terms was less than 0.05. KOBAS software was used to detect the KEGG pathways enriched with DEGs (Kanehisa et al., 2007). The standard of significantly enriched pathway is the same as GO enrichment.

## Results

### Device of transcriptome experiments and phenotypic, physiological and biochemical responses

**Figure 1:**
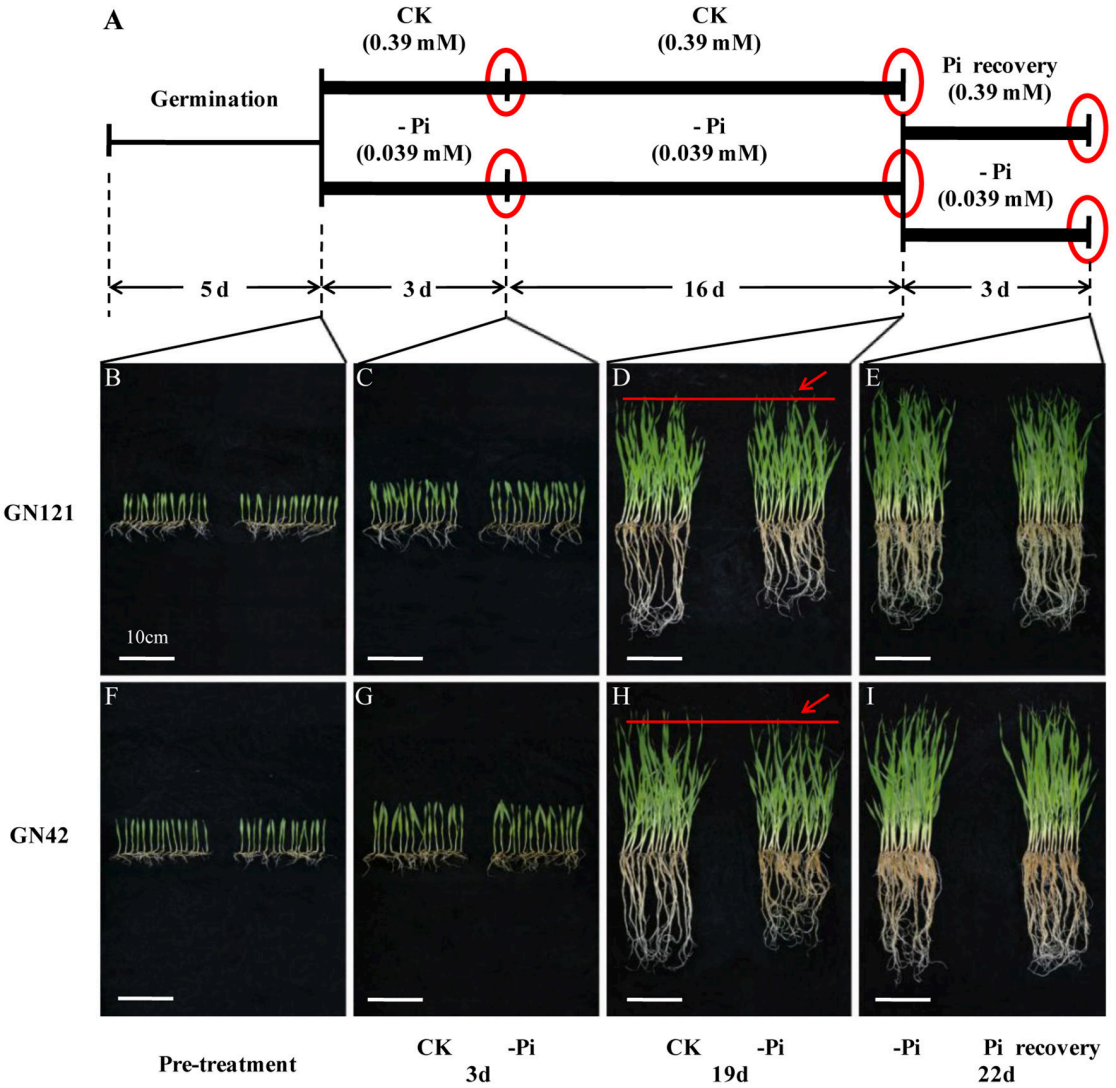
Design of experiment, and morphological changes of barley in response to phosphorus deficiency and recovery. **(A)** Illustration of experiment design. Barley seeds were sprouted in distilled water for 5 d. Uniform seedlings without endosperms were cultivated with normal (CK: 0.39 mM) and low (–Pi: 0.039 mM) phosphorus solution for 19 d. After 19 d of treatment, half of the seedlings planted in phosphorus deficiency solution were recovered with normal (0.39 mM) phosphorus for 3 d, while the other half of the seedlings were continuously maintained under phosphorus starvation to act as control. Red circles represent the three time points of sample collection. **(B–I)** Phenotypic appearance of 15 GN121 and GN42 seedlings grown at the time of pre-treatment and three key time points under different conditions. **(B,F)**, seedlings before treatment. **(C,G)**, seedlings after 3 d of treatment. **(D,H)**, seedlings after 19 d of treatment. Red lines and arrows indicate the difference of growth between CK and –Pi treatment. **(E,I)**, seedlings after 3 d of recovery and constant –Pi stress. Bar = 10 cm.

Time-course experiments were conducted for the transcriptome in order to better understand the complex mechanisms of Pi homeostasis in barley. Seedlings germinated in culture dishes for 5 d were transferred to 20 L containers and treated with normal (0.39 mM) or low (0.039 mM) phosphorus solution. Roots and leaves of the seedlings grown for 3 and 19 d were collected. After 19 d of low phosphorus treatment, half of these plants were resupplied with Pi-sufficient media (0.39 mM) for 3 d, while the other half of the plants continuously remained in –Pi conditions to serve as controls. For nutrient stress, 3 d was the critical point to distinguish between short-term and long-term treatment, based on the previous study (Secco et al., 2013). In the pre-experiment, the difference in phenotypes between control and treatment groups initially occurred at 19 d after phosphorus deficiency, which was chosen as the end point of the long-term treatment and the start point of recovery for another 3 d. In total, three time points were chosen for further study (Figure 1A).

**Figure 2:**
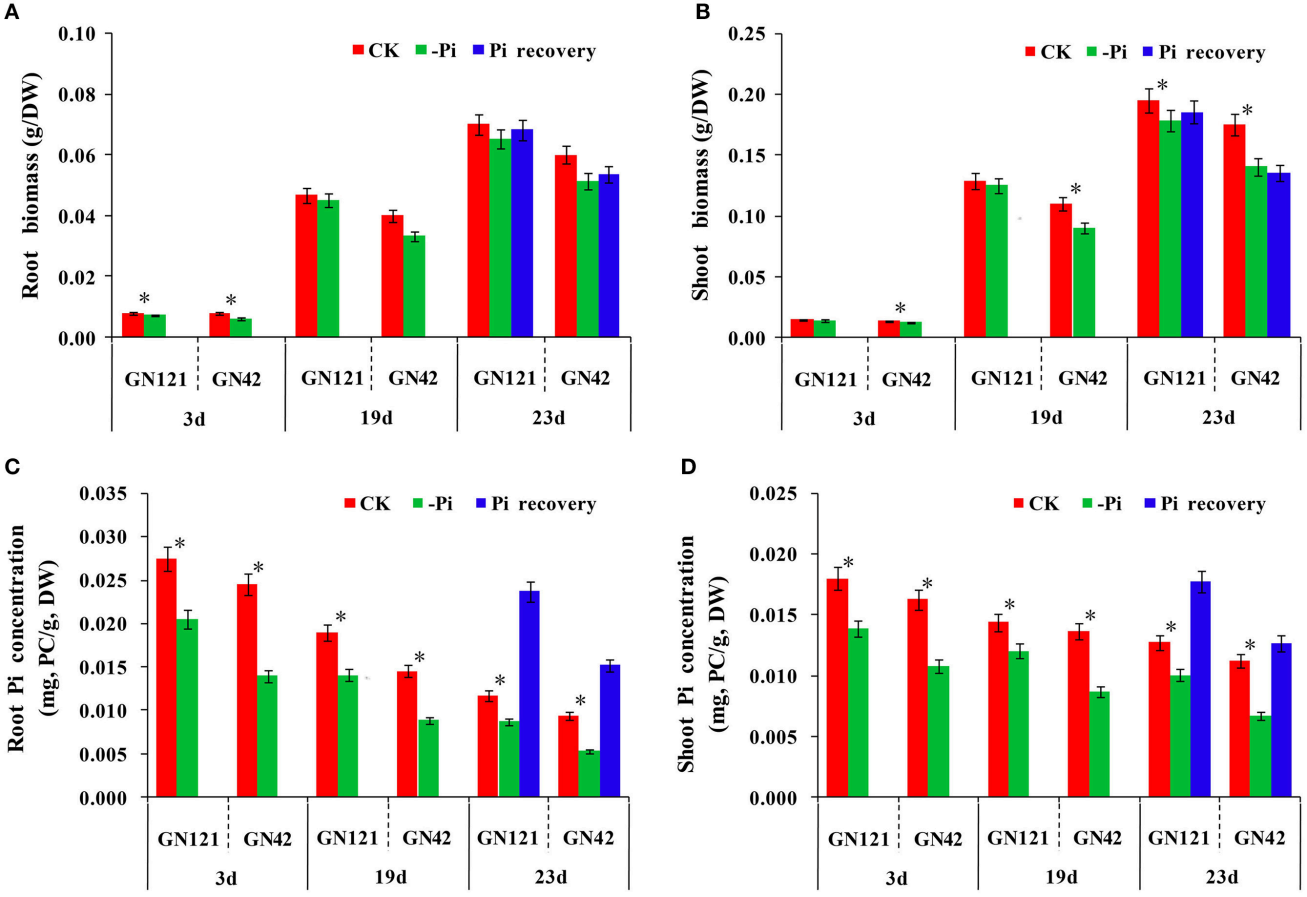
Difference of GN121 and GN42 at physiological and biochemical levels with three different treatments. **(A,B)** Biomass of roots and shoots. **(C,D)** Pi concentration of roots and shoots. Errors bars are SD and *n* = 3. ^*^indicates significant differences between CK and –Pi treatment (*P* < 0.05).

**Figure 3:**
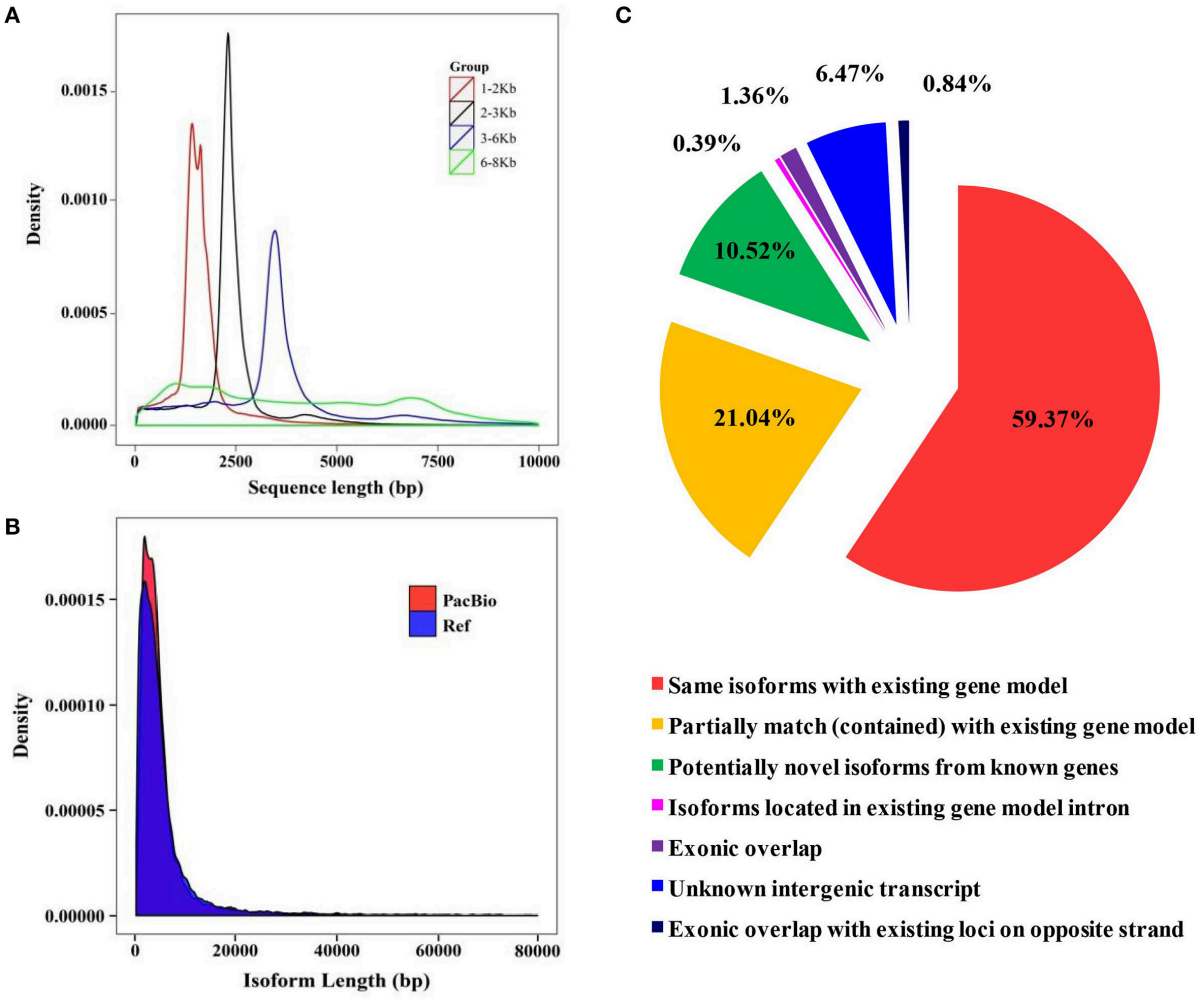
Library construction of SMRT sequencing and isoform comparison between the barley genome and full-length transcriptome. **(A)** Quality inspection of four libraries. **(B)** Isoform length comparison between the reference genome and PacBio long-reads data. **(C)** Comparison of isoforms sequences between the barley genome and full-length transcriptome.

In terms of morphological and physiological analysis, 15 seedlings of the two genotypes (GN121, low-P-tolerant and GN42, low-P-sensitive), grown in different conditions, were randomly selected for growth observation and determination of biomass and Pi concentration (Figures 1B–I, 2). There was no growth difference in roots and shoots observed by the naked eye between CK and –Pi after treating for 3 d in either GN121 or GN42, while low-P treatment caused a statistically significant reduction in the dry weight of shoots and roots for the two genotypes (Figures 1C,G). Compared with the control, the biomass of the roots and shoots of GN121 decreased by 7.96 and 3.50%, respectively, and in GN42, the biomass fell sharply by 21.79 and 12.03%, respectively (Figures 2A,B). After 19 d, root growth of GN121 and GN42 cultivated in –Pi media was significantly slower than the seedlings treated with normal phosphorus. Furthermore, shoot growth of GN42 also decreased slightly, but shoot growth of GN121 was almost the same compared with control groups (Figures 1D,H). After 19 d, the biomass of roots and shoots of GN42 showed the maximum reduction of 16.67 and 18.18%, respectively with low P levels relative to normal conditions (control), while GN121 was relatively less affected (Figures 2A,B). After 3 d of recovery, the root growth of GN121 and GN42 slightly increased, and the shoot growth of GN121 was also enhanced compared with the control group (continuous –Pi stress), while the shoot growth of GN42 was opposite to that of GN121 (Figures 1E,I). As shown in Figures 3A,B, the root biomass of GN121 and GN42 increased by 4.59% and 4.22% compared with the control, respectively. However, the shoot biomass of GN121 increased by 3.74% and that of GN42 decreased by 3.57% compared with their control groups (Figures 2A,B).

Pi concentration analysis revealed that both shoots and roots showed a gradual decrease in Pi content as the duration of the treatment increased (Figures 2C,D). Although the difference between the control group and low phosphorus treatment group was significant in both two genotypes, GN121 was much less affected by low-P than GN42. However, Pi resupply resulted in a rapid increase of the internal Pi content in both roots and shoots, and GN121 had a significantly higher shoot P concentration than GN42. Taken together, the current results clearly show that GN121 is more tolerant to low-P than GN42.

### Next generation sequencing

To analyze the effects of Pi status on the transcriptome of barley roots and leaves, we selected three time points (Figure 1A) and used three biological replicates per condition for RNA-seq, representing a total of 72 libraries. Correlation analysis results of three replications of 72 samples show that Pearson's correlation coefficient is between 0.948 and 0.989, and these values are greater than the 0.92 recommended under ideal experimental conditions (Supplemental Table 1). A dataset with 557.95 gigabases (Gb) and 3,719,505,316 raw reads were obtained in total. A number of 3,559,755,690 clean reads (Q20 > 95.09%) was generated after filtering the original data, the GC content of which was between 54.20 and 56.96%. The error rate of all clean data per sample is controlled below 0.02% (Supplemental Table 2). The amount of clean reads ranged from 39,316,198 to 61,394,388 among the 72 samples, of which between 79.97 and 90.99% mapped to the barley genome (Supplemental Table 3 and Supplemental Figure 1A). Of these mapped data, the percentage of samples that uniquely mapped to the genome was between 73.98 and 82.76%.

### PacBio Iso-Seq sequencing

Due to the short-read data and inaccurate identification of full-length splice variants from genes generated using the Illumina platform, the Pacific Biosciences Iso-Seq platform was applied to sequence the transcriptome of barley seedlings (Steijger et al., 2013; Abdel-Ghany et al., 2016). A total of 15 libraries (1–2 Kb 5, 2–3 Kb 5, 3–6 Kb 3, and 6–8 Kb 2) were constructed to eliminate bias of the instrument to the short fragment (Figure 3A). A total of 866,099 Circular Consensus (CCS) reads were obtained after filtering with SMRTLink (4.0). The length of CCS reads in all libraries was between 1,669 and 6,482 bp (Supplemental Table 4). The amount of full length reads was 662,916 (76.54% reads of total CCS reads), which contained poly-A, 5′ and 3′ primers. In total, 54,400 high-quality isoforms were identified (Supplemental Table 5). There is little difference in transcript lengths between SMRT sequencing and the known genome. However, the transcript density of SMRT sequencing was greater than the barley genome. These results showed that more transcripts were identified based on SMRT sequencing (Figure 3B and Supplemental Figure 1C). According to the results of mapping to the barley genome, a total of 59.4% of isoforms detected in SMRT sequencing were the same as with genome annotations, and 21% of all transcripts were partially mapped to the genome (Figure 3C). The barley genome was enriched with the SMRT results and used for further analysis.

### Identification of novel genes, transcription factors, fusion genes and LncRNAs

**Figure 4:**
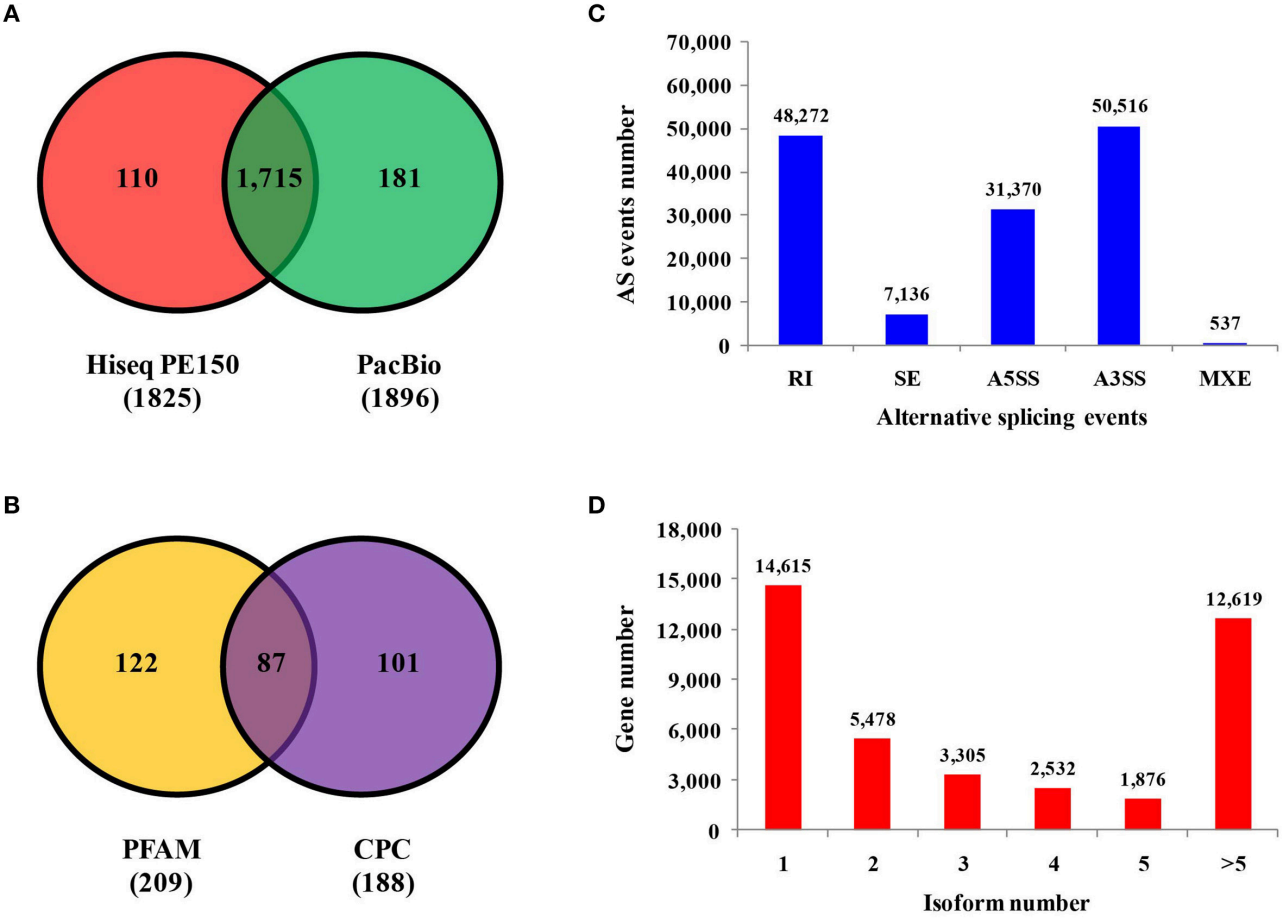
Identification of transcription factors, LncRNAs, alternative splicing events and isoform numbers based on transcriptome technologies. **(A)** Number of transcription factors sequenced on the Illumina and PacBio platforms. **(B)** Number of long non-coding RNAs analyzed by PFAM and CPC based on PacBio platform. **(C)** Number and categories of alternative splicing events based on the PacBio platform. **(D)** Number and categories of isoforms based on the PacBio platform.

Predicting novel genes may offer a new direction to further study phosphate starvation. Comparison with the barley genome, 4,656 and 691 novel genes were identified by RNA-seq and PacBio sequencing, respectively (Supplemental Figure 1B and Supplemental Tables 6, 7). The number of Pacbio was much lower than that of RNA-seq, which could be the result of long-reads length sequenced by Pacbio. Transcription factors are essential for regulation of gene expression, which need to specifically bind to certain genes. A total of 1,825 and 1,896 transcription factors were found by RNA-seq and Pacbio sequencing, respectively (Figure 4A). Of these two libraries, 1,715 transcription factors were found in both RNA-seq and Pacbio.

A total of 521 fusion genes were identified in the Pacbio library. The majority of these genes were mapped to the second chromosome, and the location of 44 fusion genes was unknown. The number of inter-chromosomal fusion genes was greater than intra-chromosomal fusion genes (Supplemental Figure 1F). Long non-coding RNA (LncRNA) is type of RNA that does not encode proteins. A total of 188 and 209 LncRNAs were found by using CPC and PFAM software, respectively, the majority of which existed in all chromosomes except the fourth and sixth chromosomes (Supplemental Figure 1E). A total of 87 LncRNAs were found by both CPC and PFAM analysis (Figure 4B).

### Detection of alternative splicing events and isoforms

Five kinds of alternative splicing (AS) events were identified by using AStalavista analysis (http://genome.crg.es/astalavista/FAQ.html). A total of 137,831 AS events were found in barley based on Pacbio reads and corresponding annotated gene models, which occurred mainly in the second, third, fifth and seventh chromosomes (Supplemental Figure 1D). The number of alternative 3′ splice sites (50,516), alternative 5′ splice sites (31,370) and retained introns (48,272) were much more than skipped exons (7,136) and mutually exclusive exons (537). The distribution of AS events is not the same as other plants, with the majority of AS events being alternative 3′ splice site events (Figure 4C). To some extent, the isoform is the result from alternative splicing. Isoform numbers are summarized from Iso-Seq reads (Figure 4D). In 14,615 genes (36.15%), only a single isoform was detected, and the number of genes with two or more isoforms was 25,810. For 12,619 genes, five or more splice isoforms were detected. Overall, Iso-Seq revealed more than 7,506 novel splice isoforms unannotated in the published version of the barley genome in this study.

### Analysis of the DEGs and qRT-PCR

**Figure 5:**
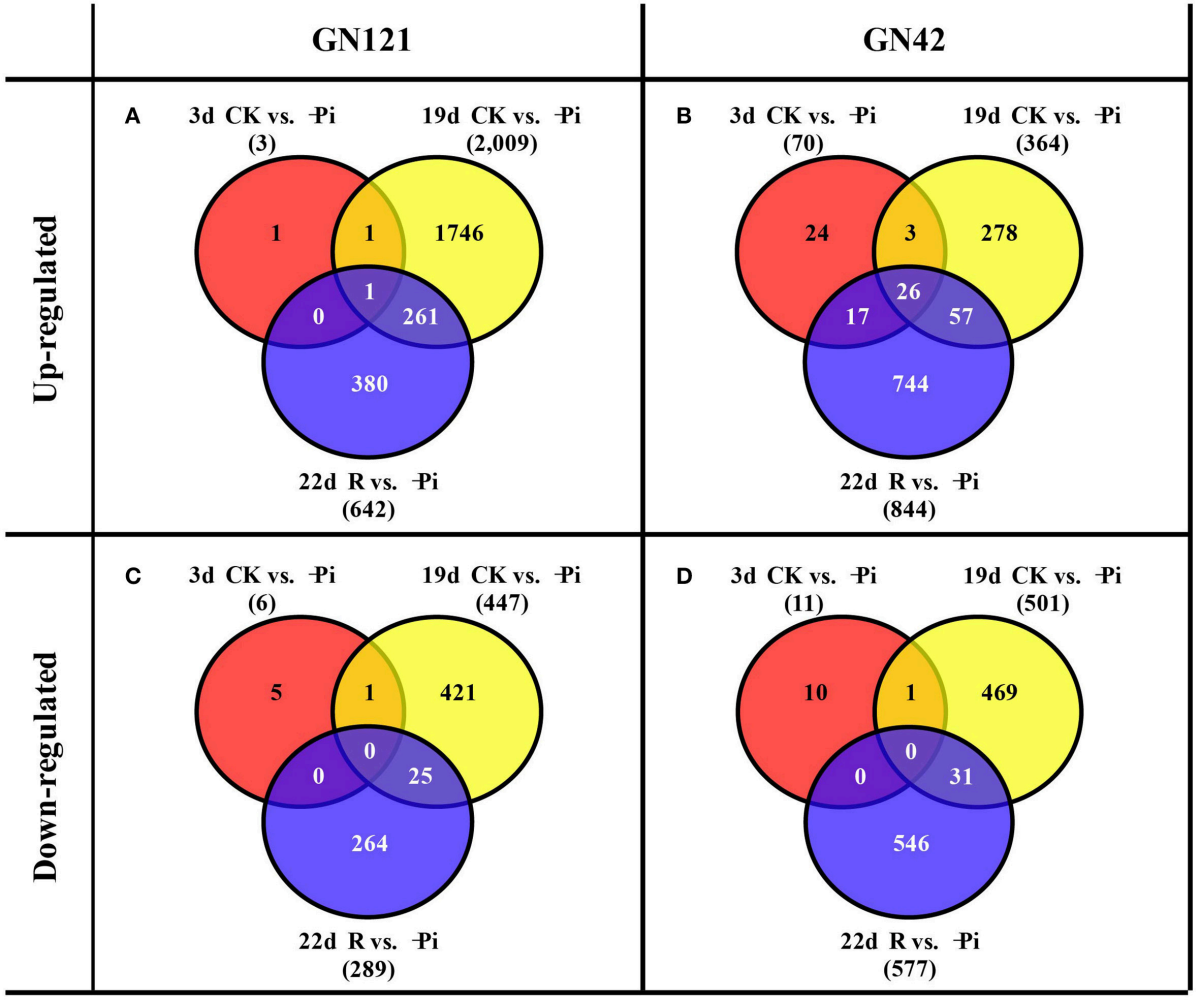
Venn diagrams of differentially expressed genes (DEGs) between the treatment and control groups in the roots of GN121 and GN42 at three time points. **(A)** Number of up-regulated genes in GN121. **(B)** Number of up-regulated genes in GN42. **(C)** Number of down-regulated genes in GN121. **(D)** Number of down-regulated genes in GN42.

DESeq software was applied to analyze the differently expression genes based on the readcount value of each transcript. Meanwhile, adjusted *P* < 0.01 were set as screening thresholds to test the significance of differences in transcript abundance. In roots, the up-regulated and down-regulated DEGs of GN121 were far fewer than those of GN42 after stressing and recovering for 3 d. A total of 2,009 up-regulated and 447 down-regulated DEGs were identified at 19 d in GN121, while the up- and down-regulated DEGs of GN42 were 364 and 501, respectively (Figures 5A–D). Interestingly, GN121 only had more up-regulated DEGs in roots than GN42 at 19 d of low phosphorus stress. As the time of stress proceeded, the number of DEGs in GN121 increased, but then decreased when resupplied with normal phosphorus, while the number of DEGs in GN42 was not affected when normal phosphorus was resupplied.

The number of up- and down-regulated DEGs of GN121 in leaves was greater than that of GN42, apart from down-regulated DEGs after 19 d of stress. As the time of stressed increased, the DEGs of GN121 continued to increase regardless of phosphorus recovery for 3 d. However, the amount of GN42 DEGs progressively increased and then declined after 3 d of resupply with control phosphorus levels (Supplemental Figure 2).

A total of 32 DEGs were identified as co-expressed genes at 3 d, 19 d and 22 d, of which 7 and 25 DEGs were in leaves and roots, respectively. The FPKM of all these co-expressed DEGs under low-P-stress at 19 d and 22 d, in both GN121 and GN42, are shown in Supplemental Table 8, although some were not differently expressed between low-P-stress and control levels. In leaves, the FPKM of seven co-expressed genes in GN121 increased under low phosphorus conditions at 22 d compared with 19 d, except for no.5 gene (HORVU7Hr1G089910), while these genes declined sharply in GN42 except no.1 (HORVU4Hr1G079600) and no.7 (HORVU5Hr1G005290). Compared with 19 d of low phosphorus treatment, 25 DEGs of roots all decreased after 22 d of stress, apart from no.4 (HORVU7Hr1G117400) and no.5 (HORVU5Hr1G062450) in GN121, and the FPKM value of GN42 DEGs was more significantly reduced than that of GN121. No co-expressed DEGs were found in leaves between GN121 and GN42, while only one co-expressed DEG was identified in roots. The co-expressed gene (no.10 HORVU3Hr1G010540) of leaves was defined as Sulfoquinovosyl transferase (SQD2), which can catalyze the final step in sulfolipid biosynthesis.

A total of 28 and 32 DEGs related to phosphorus metabolism were identified in roots and leaves, respectively. These DEGs can be divided into six categories, including genes involved in transport, transcription, lipid metabolism, metabolism, phosphorylation/dephosphorylation and miscellaneous (Lan et al., 2015). The Log_2_ (fold changes) value of root DEGs at three time points in both GN121 and GN42 are displayed in Table 1. There were no DEGs in GN121 and few DEGs in GN42 after 3 d of treatment. A large number of DEGs were identified in both GN121 and GN42 at 19 d, and the DEGs at 22 d were slightly fewer than those of 19 d. In general, the number of DEGs in GN121 was much greater than GN42, except at 3 d. The DEGs of GN121 were all up-regulated at 19 and 22 d, except for one nicotianamine synthase-related DEG at 22 d, while slightly more than half of the DEGs were up-regulated in GN42 at 19 and 22 d. In terms of transporters, more DEGs were up-regulated in GN42 at 19 d than in GN121, while no DEGs were identified in GN42 after resupplying with normal phosphorus for 3 d, contrary to the sharply down-regulated DEGs in GN121. One DEG (HORVU6Hr1G089130) defined as phosphate transporter (*PHO1;2*), which can transport Pi from roots to shoots, was up-regulated in GN121 at 22 d and GN42 at 19 d. In the transcription factors group, one SPX- (HORVU7Hr1G121090) related protein was up-regulated after stress for 19 d in GN121, while all three SPX proteins were up-regulated as early as 3 d of treatment in GN42. Three monogalactosyl-diacylglycerol (MGDG) synthases of the lipid metabolism group (HORVU0Hr1G022310, HORVU4Hr1G044140 and HORVU6Hr1G087090), which can degrade the phospholipid, were found in GN121 at 19 d, while two of these were also identified in GN42 and up-regulated at 3 or 22 d. There was one nicotianamine synthase-related DEG in the metabolism class, which can be an intermediate for the biosynthesis of mugineic acids (MAs) secreted to rhizospheres for releasing Fe. Three acid phosphatases and three chaperone proteins were identified in the phosphorylation/dephosphorylation and miscellaneous groups, respectively.

**Table 1:** List of DEGs related to phosphorus metabolism in roots.

**Gene ID**	**GN121 Log2(Fold changes)**			**GN42 Log2(Fold changes)**			**Annotation**
	**3d –Pi/CK**	**19d –Pi/CK**	**22d –Pi/Pi recovery**	**3d –Pi/CK**	**19d –Pi/CK**	**22d –Pi/Pi recovery**	
**TRANSPORTER**							
HORVU4Hr1G080350		0.89324	1.1513		1.3016		Inorganic phosphate transporter 1-2
HORVU5Hr1G110180		1.0392					Inorganic phosphate transporter 1-4
HORVU4Hr1G080730			∞		3.5395		Inorganic phosphate transporter 1-7
HORVU5Hr1G117080		0.97016	1.2663		1.8765		Inorganic phosphate transporter 1-8
HORVU0Hr1G020720			2.0918	1.4949	1.6623		Inorganic phosphate transporter 1-10
HORVU6Hr1G089130			0.87381		0.83081		Phosphate transporter PHO1-2
**TRANSCRIPTION**							
HORVU7Hr1G089910			3.0508	0.97688	1.4993	3.0085	SPX domain-containing protein 1
HORVU7Hr1G121090		1.8817	6.1861	2.133	2.3919	6.0057	SPX domain-containing protein 3
HORVU2Hr1G031400			3.437	2.1367		4.1308	SPX domain-containing protein 5
**LIPID METABOLISM**							
HORVU4Hr1G067920			1.1823			1.1639	Digalactosyldiacylglycerol synthase 2
HORVU2Hr1G070700		2.1364					Glycerophosphodiester phosphodiesterase GDPD1
HORVU3Hr1G079900		0.60715	3.1336	1.5273	1.2394	2.7952	Glycerophosphodiester phosphodiesterase GDPD1
HORVU0Hr1G022310		2.0344					Monogalactosyldiacylglycerol synthase 2
HORVU4Hr1G044140		1.9634	4.6021	3.1274	2.5118	4.6518	Monogalactosyldiacylglycerol synthase 2
HORVU6Hr1G087090		0.75801				0.67922	Monogalactosyldiacylglycerol synthase 3
**METABOLISM**							
HORVU6Hr1G032290			−5.0424			−2.0331	Nicotianamine synthase 1
HORVU2Hr1G119460					−0.82846		Pyruvate kinase
HORVU2Hr1G017300					−2.3222	−0.6887	Thioredoxin-like 1-1
HORVU2Hr1G017310					−2.3158		Thioredoxin-like 1-1
HORVU4Hr1G051400		0.96835					Thioredoxin-like 1-2
**PHOSPHORYLATION/DEPHOSPHORYLATION**							
HORVU7Hr1G018600		1.257				−0.89126	Acid phosphatase 1
HORVU7Hr1G018620		1.1427					Acid phosphatase 1
HORVU4Hr1G064360		0.78672	1.6388			1.6766	Purple acid phosphatase 17
HORVU2Hr1G112330		3.2043				1.9293	Purple acid phosphatase 22
HORVU5Hr1G042160		0.6118	1.4329			1.3098	Purple acid phosphatase 22
**MISCELLANEOUS**							
HORVU7Hr1G113530		1.5486					Chaperone protein dnaJ 8
HORVU7Hr1G116890		1.785					Chaperone protein dnaJ 11
HORVU3Hr1G014950		1.1754					Chaperone protein dnaJ 20

*–Pi, treated with low phosphorus solution. CK/Pi recovery, treated with normal phosphorus solution*.

In leaves, 14 DEGs related to phosphorus were also found in roots, which contained four DEGs in the transporter group, three DEGs in the transcription group, five DEGs in the lipid metabolism group, and two DEGs in the phosphorylation/dephosphorylation (Supplemental Table 9). Regarding transporters, one DEG (HORVU0Hr1G020720) was up-regulated after 19 d of stress and down-regulated after resupplying with normal phosphorus levels in both GN121 and GN42. Four DEGs were only down-regulated in GN121 at 22 d. Four transcription DEGs were identified and up-regulated in both GN121 and GN42. In terms of lipid metabolism and metabolism, the number of DEGs in GN121 was much greater than in GN42. Furthermore, all of these DEGs were up-regulated in GN121 at 19 d except for HORVU2Hr1G073850, HORVU2Hr1G032670, and HORVU4Hr1G061040. In the class of phosphorylation/dephosphorylation, two DEGs were down-regulated in both GN121 and GN42 under recovery conditions, and one of these was also up-regulated in GN121 at 19 d. Two DEGs of the miscellaneous group were down-regulated after resupply of normal phosphorus levels at 22 d, and the other two DEGs were down-regulated in GN42 after 19 d of stress.

Five DEGs were selected from roots and leaves for quantitative RT-PCR analysis to validate the RNA-Seq data. The results show a consistent expression trend between RNA sequencing and qRT-PCR (Supplemental Figure 3).

### GO analysis of DEGs

In order to gain a better understanding of DEG function, GO functional enrichment analysis was conducted in roots and leaves of both GN121 and GN42. The number of all root DEGs in GN121 and GN42 was 3,395 and 2,373 at three time points, and the number of all leaf DEGs was 3,053 and 2,372, respectively. These DEGs were all classified into three categories, namely biological processes, molecular functions and cellular components. In roots, the DEGs with known annotations of GN121 and GN42 could be categorized into 51 and 48 functional groups, respectively (Supplemental Figure 4). In the biological process ontology, the dominant terms in both GN121 and GN42 were “metabolic process,” “cellular process,” and “single-organism process.” Specific terms “biological phase,” and “cell killing,” were found in GN121 and GN42, respectively. The processes represented by the GO terms “cell part,” “cell,” “organelle,” “membrane,” “membrane part,” and “macromolecular complex,” accounted for the majority of the cellular components. Four terms (“synapse,” “cell junction,” “synapse part,” and “nucleoid”) were only identified in GN121. Regarding molecular function, the dominant terms in both GN121 and GN42 were “binding,” “catalytic activity,” “transporter activity,” “structural molecule activity,” and “nucleic acid binding transcription factor activity.” One term “metallochaperone activity” was only found in GN42.

There were 54 and 48 functional groups of leaves formed in GN121 and GN42 according to their DEGs, respectively (Supplemental Figure 5). The processes represented by the GO terms “metabolic process,” “cellular process,” and “single-organism process,” accounted for the dominant terms of biological processes in GN121 and GN42. Two terms (“biological phase” and “cell aggregation”) were specifically found in GN121. Regarding molecular function, the majority of terms in both GN121 and GN42 were “binding,” “catalytic activity,” “transporter activity,” “structural molecule activity,” and “nucleic acid binding transcription factor activity.” One term “metallochaperone activity” was only identified in GN121. With respect to cellular component ontology, the majority of terms in both GN121 and GN42 were “cell,” “cell part,” “membrane,” “organelle,” “membrane part,” and “macromolecular complex.” The typical terms “synapse part,” “synapse,” “extracellular matrix component,” and “nucleoid” were found in GN121, and “cell junction” was only identified in GN42.

### KEGG enrichment analysis

For illustrating the reaction between DEGs identified from RNA-seq and the known genes in the metabolism pathway, KEGG pathway enrichment analysis was carried out. In roots, 2,233 DEGs of GN121 were assigned to 115 KEGG pathways, and 1,593 DEGs of GN42 were assigned to 110 KEGG pathways (Supplemental Figure 6). Four pathways (“metabolic pathways,” “biosynthesis of secondary metabolites,” “ribosome,” and “phenylpropanoid biosynthesis”) were the most abundant in GN121, while two pathways (“metabolic pathways” and “biosynthesis of secondary metabolites”) were plentiful in GN42. Ten pathways closely related to phosphorus were identified, including “plant hormone signal transduction,” “starch and sucrose metabolism,” “brassinosteroid biosynthesis,” “glycolysis/gluconeogenesis,” “glycerolipid metabolism,” “pentose phosphate pathway,” “inositol phosphate metabolism,” “glycerophospholipid metabolism,” “citrate cycle,” and “pyruvate metabolism.” Among these 10 pathways, the number of DEGs of GN121 was greater than that of GN42 except for three pathways (“citrate cycle,” “glycolysis/gluconeogenesis,” and “pentose phosphate pathway”). In addition, there were seven (“regulation of autophagy,” “non-homologous end-joining,” “limonene and pinene degradation,” “C5-branched dibasic acid metabolism,” “folate biosynthesis,” “glycosylphosphatidylinositol(GPI)-anchor biosynthesis,” and “basal transcription factors”) and two (“glycosphingolipid biosynthesis—globo series” and “proteasome”) pathways found in GN121 and GN42, respectively.

In total, 2,313 and 1,514 DEGs in leaves encoding various enzymes were investigated for KEGG pathway enrichment in GN121 and GN42, respectively. There were 117 KEGG pathways identified in GN121 and GN42, respectively (Supplemental Figure 7). Regarding the 10 phosphorus pathways mentioned above, there were fewer DEGs in GN121 compared to GN42 in the “plant hormone signal transduction” pathway. Two typical pathways (“C5-branched dibasic acid metabolism” and “glycosylphosphatidylinositol-anchor biosynthesis”) were found in GN121 and another two pathways (“glycosaminoglycan degradation” and “folate biosynthesis”) were identified in GN42.

### Construction of the putative model

**Figure 6:**
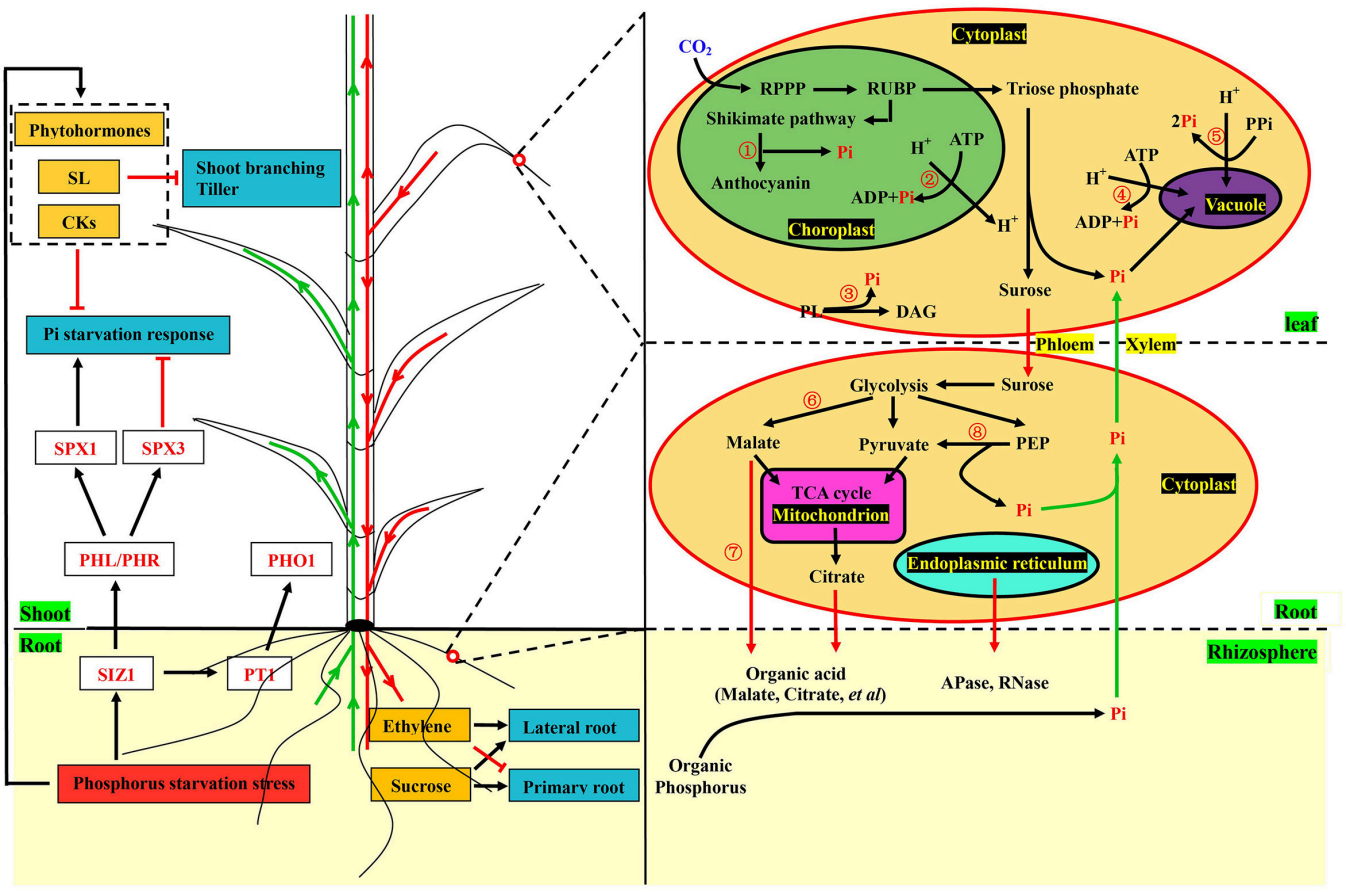
Illustration of key components involved in phosphorus starvation metabolic pathway in barley. Plant hormones and phosphorus transporters, acting as sensors and messengers, quickly responded to the changes of external phosphorus levels. The synthesis of strigolactones and cytokinin suppressed shoot branching and the Pi starvation response, respectively. Expression of phosphorus transporters were regulated by SUMO E3 ligase SIZ1, which also positively regulated the response of Pi deficiency through the MYB transcription factor PHL/PHR and SPX1. Phloem-mobile sucrose and ethylene could regulate the changes of root system architecture (RSA). In the cells of leaves, experiments were performed at physiological and molecular levels. Anthocyanin synthesis (①) was conducted after the shikimate pathway and Pi was released at the same time. Phospholipids (③) were degraded into Pi and replaced by glycolipids and sulfolipids. Molecules (②, ④, and ⑤) containing phosphorus were also degraded into Pi. In the root cells, organic acids (⑥ and ⑦) and enzymes including acid phosphates and RNase were produced and excreted into rhizospheres to solubilize compounds containing phosphorus. Phosphoenolpyruvate (⑧) was catalyzed into pyruvate and Pi by kinase. Pi and other metabolites were transported from roots to shoots through the xylem (green arrows), while sucrose and ethylene were conveyed from shoots to roots by the phloem in red arrows, which also indicate that transportation was conducted from roots to rhizospheres. Black arrows and red terminated lines represent positive regulation and suppressed expression, respectively. Key genes, transcription factors and Pi are marked in red font. The yellow and dark cyan boxes represent metabolites and responses of Pi deficiency, respectively. The red box shows the condition of the external environment. The dotted box represents the plant hormones. The words with green background express the tissues of plant and rhizosphere. Words with yellow background represent xylem and phloem. The yellow ovals show the cells in the roots and leaves. In the cell, the green oval represents the chloroplast. The purple oval represents the vacuole. The pink oval represents the mitochondria. The light cyan oval represents the endoplasmic reticulum. The yellow words with black background represent the organelles and cytoplast. The words with black font express metabolic intermediates and organic phosphorus except RPPP, shikimate pathway, glycolysis and the TCA cycle. SL, Strigolactone; CK, Cytokinins; Pi, Inorganic phosphorus; SPX1/3, Transcription factors of SPX family; PHL/PHR, MYB-type transcription factors; SIZ1, SUMO E3 ligase; PT1/PHO1, Phosphorus transporters; RPPP, Reductive pentose phosphate pathway; RUBP, Ribulose 1,5-bisphosphate carboxylase/oxygenase; ATP, Adenosine triphosphate; ADP, Adenosine diphosphate; PL, Phospholipid; DAG, Diacylglycerol; PPi, Inorganic pyrophosphate; PEP, Phosphoenolpyruvate; TCA cycle, Tricarboxylic acid cycle.

To better understand the complex response processes, we constructed a theoretical model based on pivotal data at the transcriptional level (Figure 6). Sensing phosphorus limitation, and signal transduction thereof were the first steps in responding to the change in this specific environmental nutrient (Zhang et al., 2014). Phosphorus transporters and plant hormones are important sensors and messengers involved in these processes (Chiou and Lin, 2011; Zhang et al., 2014). Although specific phosphorus transporters (PHT1), acting as sensors of low phosphorus have not been identified in barley, *PHO1* and *Pho84* have been shown to be PHT1 in *Arabidopsis* and yeast (Hamburger et al., 2002; Popova et al., 2010; Ham et al., 2018). Strigolactones (SLs), which are plant hormones, are long-distance signaling molecules from roots to shoots for inhibiting tiller and regulating root system architecture (RSA) (Umehara et al., 2010; Ruyter-Spira et al., 2011). In addition, anthocyanin was accumulated and used as a significant visual feature under the low phosphorus stress (Li et al., 2014). In the cell, sucrose synthesis and degradation of molecules containing phosphorus occur during phosphorus starvation. Meanwhile, organic acids and hydrolases are produced and secreted into rhizospheres to release the phosphorus (Zhang et al., 2014). All these biological metabolic processes related to phosphorus were detected in our study, and most of the vital genes involved in these processes were up-regulated in GN121 and down-regulated in GN42 under the phosphorus deficiency (Supplemental Figure 8). Our results suggest that significant differences of phosphate metabolism between the two different genotypes did indeed exist, and we provided the scientific basis for further studies on phosphorus efficiency.

## Discussion

Phosphorus, one of the indispensable elements for life, usually exists as a component in biomolecules (Van Mooy et al., 2015). Crop production has been seriously affected by a deficiency in available phosphorus in the soil. The investment of phosphorus fertilizer has, however, alleviated this serious problem, but the ensuing environmental pollution is becoming more and more acute (Tong et al., 2017). Based on the current status, selection of low-P tolerant genotypes is one of the most efficient solutions to breed new crop varieties for improving crop yield and meet the demands of increasing populations. Two barley genotypes (GN121 and GN42) with contrasting phosphorus efficiency were further identified by morphological and physiological characteristics. Subsequently, molecular mechanisms involved in the diversity of responses to P deficiency in GN121 and GN42 were studied using the transcriptome technique. In the present study, three time points (3, 19, and 22 d) were selected for investigating physiological and molecular mechanisms. Secco et al. (2013) reported that sensing a lack of Pi, and signal transduction thereof, was either instantaneous or occurred within 3 d of low phosphorus treatment, through studying transcription profiling in rice (Secco et al., 2013). These results were consistent with morphological observations of our study at 3 d. In two other studies of nutrient stress, hour 6 and hour 48 were chosen as time points. Although a large number of DEGs were identified, the majority of which were relative to nonspecific responses (Zeng et al., 2014; Quan et al., 2016). Selection of the day 19 relied on the occurrence of differences between CK and low phosphorus treatment in the preliminary experiment. This time point was also in line with the previous study (Secco et al., 2013). Recovery for 3 d was aligned with the first time point for analyzing the differences at a morphological and molecular level, and the continuous low phosphorus stress was used as the control.

### Analysis of morphological and physiological changes

Previous study reported that growth of shoots and roots declined under Pi-limiting conditons (Secco et al., 2013). Other investigations also showed biomass and dry weight could be evaluation indexes for nutrition stress (Broadley et al., 2004; Hermans et al., 2006). Pi concentration in plants can reflect the ability of absorption and potential redistribution under different phosphate conditions. In the present study, biomass and Pi concentration of roots and shoots in both GN121 and GN42 decreased under the low-P treatment compared with the control group, while biomass and Pi concentration increased in both genotypes at 22 d, with the exception of shoot biomass of GN42 (Figure 2). The trend of these parameters is consistent with previous studies (Du et al., 2016; Li et al., 2016). These results indicate that GN121 had greater capability of P absorption and translocation than GN42.

### Character of genome enriched with SMRT reads and analysis of LncRNAs and AS events

In the current study, the percentage mapped to the known genome of next generation and SMRT sequencing reached up to 90.99 and 80.4%, respectively, which could have mainly resulted from genes editing after transcription, the material used in the transcriptome profiling and the sequencing technology. However, information of the enriched genome was more than that of the known genome. The information on transcripts was directly obtained without splicing the fragments (Minoche et al., 2015; Xu et al., 2015). A large number of new genes and extrons were identified for researching gene function and structure, which could provide new aspects for improving the phosphorus efficiency.

LncRNA is one kind of RNAs, which could not been translated into polypeptides. Nevertheless, expression, and transcriptional regulation of genes were significantly affected by them (Batista and Chang, 2013; Iyer et al., 2015). We firstly identified LncRNAs in barley under phosphorus starvation and recovery conditions, but their function remains unknown. AS events is one of key elements for diversity of genes and proteins in eukaryotes (Gao et al., 2016). Although many AS events were obtained in the current study, different mechnisam of PUE mediated by which between GN121 and GN42 needs to be further investigated. Based on these results, future research on structure and function of these LncRNAs and AS events is imperative and helpful to better understand PUE at the molecular level.

### Sensing of phosphorus starvation signals in barley

In order to lay a solid foundation for further studies at the molecular level, 72 short-read NGS libraries and one long-read SMRT library were constructed for the first time from roots and leaves in barley. Accurate full-length sequences were generated from SMRT reads corrected with the Illumina data to avoid mis-annotations of genes for phosphorus metabolism analysis.

Previous study reported that a root cell membrane-localized sensor and an intracellular sensor are thought to sense changes in external Pi concentration and internal nutrient status, respectively (Zhang et al., 2014). The Pi transporter (*pho84*) was firstly reported as a Pi sensor in yeast (Giots et al., 2003). According to these reports, phosphate transporters (PTs) can work as sensors too (Zhang et al., 2014). In our study, five DEGs defined as PTs were identified in roots (Table 1). After 3 d of treatment, *PT1-10* was only found in GN42. The majority of PTs were up-regulated in both GN121 and GN42 at 19 d, and all were down-regulated, except for HORVU5Hr1G110180, in GN121 when supplied with normal Pi levels contrary to no PTs in GN42. As phosphate sensors, expression of these PTs in GN121 was later and more short-lived than that of GN42, which indicated that normal growth of GN121 was affected to a lesser extent compared to GN42 under different external phosphorus concentrations. These PTs were up-regulated unless there was a low available internal phosphate content, and dramatically down-regulated when supplied with phosphorus in GN121. This result was different with other investigations related to nutrition starvation (Zeng et al., 2014; Quan et al., 2016).

Plant hormones can also sense Pi stress signaling and regulate root system architecture and shoot growth. Ethylene synthesis was improved to enhance lateral root elongation and arrest primary root growth under conditions of nutrient deficiencies, including Pi (Zhu et al., 2017). The enzyme, 1-aminocyclopropane-1-carboxylate oxidase (ACO), involved in the second step of ethylene synthesis, was up-regulated under Pi deficiency (García et al., 2015). Five DEGs, defined as ACO, were also up-regulated in both GN121 and GN42, while the fold-change of GN121 was much greater than GN42. Meanwhile, expression of these genes was continuously up-regulated in GN121 as the stress time progressed. In previous studies, strigolactone (SL) was reported as an early host plant recognition signal and shoot branching inhibitor in plants (Gomez-Roldan et al., 2008). The previous study found that SL synthesis increased under Pi stress (Czarnecki et al., 2013). Strigolactone esterase (D14), a component of hormone signaling, participates in the conversion of SL to the bioactive form (Arite et al., 2009). Two D14 DEGs were identified and the up-regulated trend of GN121 was greater than that of GN42 under Pi stress compared with normal Pi levels. This result was confirmed when observing the barley phenotype. The tiller number in different conditions of GN121 was greater than that in GN42. All these results demonstrate that GN121 growth was better than GN42 under low phosphorus conditions.

### Pi transport by PHO1

PHO1 is one of the most important gene families for Pi transport from roots to shoots (Khan et al., 2014). *PHO1;2*, one member of the PHO1 family, was expressed in the vascular bundles (Jabnoune et al., 2013; Wu et al., 2013). However, Muehe et al. (2014) reported that *PHO1;2* expression in roots was higher than that in shoots (Muehe et al., 2014). In our study, *PHO1;2* was also up-regulated to a greater extent in roots than in leaves, and the up-regulated expression of *PHO1;2* under low Pi treatment in shoots was only evident in GN121, while there was no change in *PHO1;2* expression in GN42. Another phosphate transporter, *PHO1;3*, was found to be expressed in leaves prior to expression in roots (Hamburger et al., 2002). Previous studies reported that *PHO1;3* was also induced in roots by Zn deficiency (Wu et al., 2013; Khan et al., 2014). In the present study, *PHO1;3* was mainly up-regulated under poor Pi conditions in leaves, and the expression level was very low. Expression of these two genes provides the facility for root-to-shoot Pi transport in GN121.

### Pi metabolism

In addition to programmed cell death, cell killing is another way to induce cell death. Previous transcriptome studies related to abiotic stress were conducted, and some genes were identified by GO analysis, which were involved in cell killing (Liu et al., 2015; Yan et al., 2016). These results indicate that the expression of genes involved in cell killing is one of the mechanisms for plants to adapt to adverse situations. GO analysis in the present study showed a small number of cell killing genes were also enriched (GO: 0001906). Meanwhile, expression of these genes was a little higher in GN42 than in GN121. In roots, some of these genes were only enriched in GN42 at 22 d. Genes of GN121 in leaves were found at 22 d, while in GN42 they were enriched as early as 19 d. This demonstrated that GN42 was affected more severely than GN121 in response to low-P stress.

KEGG enrichment analysis was conducted on all DEGs in both roots and leaves of GN121 and GN42. Two metabolic pathways, folate biosynthesis (bdi00790) and limonene and pinene degradation (bdi00903), were only enriched in roots of GN121 at 19 d. Jiang et al. (2013) reported that the folate biosynthesis pathway is important for nitrate utilization (Jiang et al., 2013). Raven (2015) reported that the energy of N and P assimilation compete with each other (Raven, 2015). These two studies indicate that the decrease of N content in plants could indirectly result in the decline of folate biosynthesis and an increase of P accumulation. In this study, only one gene, enriched in the folate biosynthesis pathway, was down-regulated in GN121 under low-P stress at 19 d compared with the control group. This shows that GN121 was weakly affected by Pi deficiency. In a recent study, limonene and pinene degradation was reported as a stress defense mechanism process, which was significantly reinforced under abiotic stress (Tian et al., 2017). Only one gene was enriched in this pathway in the present study, and up-regulated under the condition of Pi starvation. This result demonstrates a series of defense mechanisms in GN121 were induced to adapt to low-P stress. Phospholipids, combined with one-third organic P of leaves, were replaced with galactolipids and sulfolipids under Pi-limiting conditions (Shemi et al., 2016; Angkawijaya et al., 2017). Glycerolipids, one of the galactolipids, increase under low-P stress (Siebers et al., 2015). In the present study, 22 and 21 genes were enriched in the glycerolipid metabolism pathway in GN121 and GN42, respectively. A total of 16 of these genes were co-expressed in the two genotypes, most of which were more highly expressed in GN121 than in GN42 at the three time points. The data indicates that glycerolipid metabolic activity increased in GN121.

### Transcriptional regulation of Pi metabolism

SPX domain proteins were reported to participate in Pi homeostasis (Wang Z. et al., 2014). Among these proteins, SPX1 negatively regulates Pi starvation responses by inhibiting the *PHR1* activity (Jung et al., 2018). In *Arabidopsis*, SPX1 was also observed to positively regulate phosphate-starvation-inducible genes (Duan et al., 2008). In this study, SPX1 was identified in leaves and roots of both GN121 and GN42. SPX1 was expressed in GN42 as early as 3 d, while SPX1 in GN121 was only found in roots at 22 d treatment and was slightly up-regulated in leaves at 19 d. SPX1 was dramatically down-regulated in both GN121 and GN42 once the external Pi level returned to normal. SPX3, another member of the SPX family, was reported to be involved in maintaining Pi homeostasis (Bucher and Fabianska, 2016). SPX3 was identified as a differently-expressed gene in both GN121 and GN42 at 19 and 22 d. In addition, SPX3 was only up-regulated in roots of GN42 at 3 d. The change of SPX3 expression at three time points was similar to SPX1. These results indicate the response of GN121 to external low–Pi levels was later and more rapid than GN42.

## Conclusions

Two genotypes with contrasting Pi efficiency (GN121 and GN42) were used for analyzing molecular mechanisms of PUE by high-throughput Next Generation Sequencing (NGS) and full-length transcriptome techniques. A putative model and heatmaps of key genes were built to show the differences between GN121 and GN42 for illustrating the adaptive mechanisms of plants in response to poor phosphorus. The results indicated that GN121 was much more tolerant to external low–Pi stress than GN42. Moreover, two transcriptome libraries related to Pi starvation and recovery were developed for the first time. All these findings improve our knowledge of PUE at the molecular level and have laid a solid foundation for further studies and improving the phosphorus efficiency of crops.

## Author contributions

HW and XS: experimental design. PR and YM: conducted the experiment and wrote the paper. PR, YM, YL, ES, JW, LY, and KY: analysis of all data. BL and XM: correction of the paper critically.

### Conflict of interest statement

The authors declare that the research was conducted in the absence of any commercial or financial relationships that could be construed as a potential conflict of interest.

## Supplementary material

Supplemental Figure 1 Annular appearance of data comparison between the barley genome and PacBio sequencing. Different colors express different numbers (number ≤ 10 color = deep red, number < 20 color = red, number < 30 color = light red, number < 40 color = deep yellow, number < 50 color = yellow, number < 60 color = light yellow, number < 70 color = light green, number < 80 color = green, number < 90 color = deep green, number < 100 color = light blue, number < 110 color = blue, number < 120 color = deep blue, number >= 120 color = black). **(A)** Chromosomes of the barley genome. **(B)** Gene distribution of the genome and PacBio sequencing. **(C)** Transcript distribution of the genome and PacBio sequencing. **(D)** Allocation of alternative splicing events on chromosomes. **(E)** Allocation of long non-coding RNAs on chromosomes. **(F)** Allocation of fusion genes on chromosomes. Black lines represent intra-chromosome fusion genes. Orange lines represent inter-chromosome fusion genes.

Supplemental Figure 2 Venn diagrams of differentially expressed genes (DEGs) between the treatment and control groups in the leaves of GN121 and GN42 at three time points. **(A)** Number of up-regulated genes in GN121. **(B)** Number of up-regulated genes in GN42. **(C)** Number of down-regulated genes in GN121. **(D)** Number of down-regulated genes in GN42.

Supplemental Figure 3 Quantitative reverse-transcription PCR validation of differentially expressed genes in roots and leaves. Black columns represent FPKM based on the Illumina platform. The red broken lines represent relative expression quantity tested by qRT-PCR. The bars represent SE (*n* = 3). **(A)** Expression levels of five differentially expressed genes based on RNA-seq and qRT-PCR in roots. **(B)** Expression levels of five differentially expressed genes based on RNA-seq and qRT-PCR in leaves.

Supplemental Figure 4 Gene Ontology (GO) analysis of differentially expressed genes in the roots of GN121 and GN42. Red columns represent the biological process (BP). Green columns represent the cellular component (CC). Blue columns represent the molecular function (MF). **(A)** Gene number of GO enrichment terms in the roots of GN121. **(B)** Gene number of GO enrichment terms in the roots of GN42.

Supplemental Figure 5 Gene Ontology (GO) analysis of differentially expressed genes in the leaves of GN121 and GN42. Red columns represent the biological process (BP). Green columns represent the cellular component (CC). Blue columns represent the molecular function (MF). **(A)** Gene number of GO enrichment terms in the leaves of GN121. **(B)** Gene number of GO enrichment terms in the leaves of GN42.

Supplemental Figure 6 KEGG analysis of differentially expressed genes in the roots of GN121 and GN42. **(A)** Gene number of KEGG enrichment terms in the roots of GN121. **(B)** Gene number of KEGG enrichment terms in the roots of GN42.

Supplemental Figure 7 KEGG analysis of differentially expressed genes in the leaves of GN121 and GN42. **(A)** Gene number of KEGG enrichment terms in the leaves of GN121. **(B)** Gene number of KEGG enrichment terms in the leaves of GN42.

Supplemental Figure 8 Heat maps of key differentially expressed genes involved in the phosphorus metabolic pathway at three time points in roots and leaves (CK/R, normal phosphorus level. –Pi, low phosphorus level). **(A)** Heat map of DEGs that participated in phosphorus metabolism of roots. **(B)** Heat map of DEGs that participated in phosphorus metabolism of leaves.

Supplemental Table 1 Correlation analysis of 72 samples.

Supplemental Table 2 Quality evaluation of data sequenced on the Illumina platform.

Supplemental Table 3 Comparison of data between the next generation sequencing and the barley genome.

Supplemental Table 4 Output of insert reads sequenced on the PacBio platform.

Supplemental Table 5 Results of filtering the raw data obtained from the PacBio platform.

Supplemental Table 6 Prediction of novel genes based on the Illumina platform.

Supplemental Table 7 Prediction of novel genes based on the PacBio platform.

Supplemental Table 8 List of the co-expressed differentially expressed genes between GN121 and GN42 in roots and leaves. List of the 32 co-expressed differentially expressed genes (DEGs) between GN121 and GN42 at three time points in roots and leaves (–Pi 19d, low phosphorus treatment for 19 d. –Pi 22d, low phosphorus treatment for 22 d.).

Supplemental Table 9 List of differentially expressed genes related to phosphorus metabolism in leaves. List of DEGs related to phosphorus metabolism in leaves (–Pi, treated with low phosphorus solution. CK/Pi recovery, treated with normal phosphorus solution).

Supplemental Table 10 Primers for quantitative reverse-transcription PCR.

## Abbreviations

P: phosphorus
Pi: inorganic phosphorus
PUE: phosphorus use efficiency
SMRT: Single-molecule real-time
CCS: circular-consensus
PCR: polymerase chain reaction
AS: alternative splicing
FPKM: expected number of Fragments Per Kilobase of transcript sequence per Millions base pairs sequenced
DEGs: differentially expressed genes
qRT-PCR: quantitative real-time polymerase chain reaction
GO: Gene Ontology
KEGG: Kyoto Encyclopedia of Genes and Genomes
LP: low phosphorus
LncRNA: Long non-coding RNA
SQD: Sulfoquinovosyl transferase
MGDG: monogalactosyl-diacylglycerol
MA: mugineic acid
NGS: next-generation sequencing
PT: phosphate transporter
ACO: 1-aminocyclopropane-1-carboxylate oxidase
SL: strigolactone
D14: Strigolactone esterase
N: nitrate.

## References

[B1] Abdel-Ghany S. E. Hamilton M. Jacobi J. L. Ngam P. Devitt N. Schilkey F.. (2016). A survey of the sorghum transcriptome using single-molecule long reads. Nat. Commun. 7:11706. 10.1038/ncomms11706 27339290 PMC4931028

[B2] Abel S. (2017). Phosphate scouting by root tips. Curr. Opin. Plant Biol. 39, 168–177. 10.1016/j.pbi.2017.04.016 28527590

[B3] Anders S. Huber W. (2012). Differential Expression of RNA-Seq Data at the Gene Level-the DESeq Package. Heidelberg: European Molecular Biology Laboratory (EMBL).

[B4] Anders S. Pyl P. Huber W. (2015). HTSeq–a Python framework to work with high-throughput sequencing data. Bioinformatics 31, 166–169. 10.1093/bioinformatics/btu638 25260700 PMC4287950

[B5] Andersson H. Bergström L. Djodjic F. Ulén B. Kirchmann H. (2013). Topsoil and subsoil properties influence phosphorus leaching from four agricultural soils. J. Environ. Qual. 42, 455–463. 10.2134/jeq2012.0224 23673838

[B6] Angkawijaya A. E. Nguyen V. C. Nakamura Y. (2017). Enhanced root growth in phosphate-starved Arabidopsis by stimulating de novo phospholipid biosynthesis through the overexpression of LYSOPHOSPHATIDIC ACID ACYLTRANSFERASE 2 (LPAT2). Plant Cell Environ. 40, 1807–1818. 10.1111/pce.12988 28548242

[B7] Arite T. Umehara M. Ishikawa S. Hanada A. Maekawa M. Kyozuka J.. (2009). d14, a strigolactone-insensitive mutant of rice, shows an accelerated outgrowth of tillers. Plant Cell Physiol. 50, 1416–1424. 10.1093/pcp/pcp091 19542179

[B8] Batista P. J. Chang H. Y. (2013). Long noncoding RNAs: cellular address codes in development and disease. Cell 152, 1298–1307. 10.1016/j.cell.2013.02.012 23498938 PMC3651923

[B9] Benjamini Y. Hochberg Y. (1995). Controlling the false discovery rate: a practical and powerful approach to multiple testing. J. R. Stat. Soc. Ser. B, 57, 289–300.

[B10] Broadley M. R. Bowen H. C. Cotterill H. L. Hammond J. P. Meacham M. C. White P. J.. (2004). Phylogenetic variation in the shoot mineral concentration of angiosperms. J. Exp. Bot. 55, 321–336. 10.1093/jxb/erh002 14739259

[B11] Bucher M. Fabianska I. (2016). Long-sought vacuolar phosphate transporters identified. Trends Plant Sci. 21, 463–466. 10.1016/j.tplants.2016.04.011 27160805

[B12] Byrne S. L. Foito A. Hedley P. E. Morris J. A. Stewart D. Barth S.. (2010). Early response mechanisms of perennial ryegrass (*Lolium perenne*) to phosphorus deficiency. Ann. Bot. 107, 243–254. 10.1093/aob/mcq234 21148585 PMC3025732

[B13] Chien P. S. Chiang C. B. Wang Z. Chiou T. J. (2017). MicroRNA-mediated signaling and regulation of nutrient transport and utilization. Curr. Opin. Plant Biol. 39, 73–79. 10.1016/j.pbi.2017.06.007 28668626

[B14] Chiou T. J. Lin S. I. (2011). Signaling network in sensing phosphate availability in plants. Annu. Rev. Plant Biol. 62, 185–206. 10.1146/annurev-arplant-042110-103849 21370979

[B15] Clarholm M. Skyllberg U. Rosling A. (2015). Organic acid induced release of nutrients from metal-stabilized soil organic matter–the unbutton model. Soil Biol. Biochem. 84, 168–176. 10.1016/j.soilbio.2015.02.019

[B16] Consortium I. B. G. S. (2012). A physical, genetic and functional sequence assembly of the barley genome. Nature 491:711 10.1038/nature11543 23075845

[B17] Czarnecki O. Yang J. Weston D. J. Tuskan G. A. Chen J. G. (2013). A dual role of strigolactones in phosphate acquisition and utilization in plants. Int. J. Mol. Sci. 14, 7681–7701. 10.3390/ijms14047681 23612324 PMC3645710

[B18] Ding W. Wang Y. Fang W. Gao S. Li X. (2016). TaZAT8, a C2H2-ZFP type transcription factor gene in wheat, plays critical roles in mediating tolerance to Pi deprivation through regulating P acquisition, ROS homeostasis and root system establishment. Physiol. Plant. 158, 297–311. 10.1111/ppl.12467 27194419

[B19] Du H. Yu Y. Ma Y. Gao Q. Cao Y. Chen Z.. (2017). Sequencing and de novo assembly of a near complete indica rice genome. Nat. Commun. 8:15324. 10.1038/ncomms15324 28469237 PMC5418594

[B20] Du Q. Wang K. Xu C. Zou C. Xie C. Li W. X.. (2016). Strand-specific RNA-Seq transcriptome analysis of genotypes with and without low-phosphorus tolerance provides novel insights into phosphorus-use efficiency in maize. BMC Plant Biol. 16:222. 10.1186/s12870-016-0903-4 27724863 PMC5057381

[B21] Duan K. Yi K. Dang L. Huang H. Wu W. Wu P. (2008). Characterization of a sub-family of Arabidopsis genes with the SPX domain reveals their diverse functions in plant tolerance to phosphorus starvation. Plant J. 54, 965–975. 10.1111/j.1365-313X.2008.03460.x 18315545

[B22] Florea L. Song L. Salzberg S. L. (2013). Thousands of exon skipping events differentiate among splicing patterns in sixteen human tissues. F1000Research 2. 10.12688/f1000research.2-188.v1 24555089 PMC3892928

[B23] Gao Y. Wang J. Zheng Y. Zhang J. Chen S. Zhao F. (2016). Comprehensive identification of internal structure and alternative splicing events in circular RNAs. Nat. Commun. 7:12060. 10.1038/ncomms12060 27350239 PMC4931246

[B24] García M. J. Romera F. J. Lucena C. Alcántara E. Pérez-Vicente R. (2015). Ethylene and the regulation of physiological and morphological responses to nutrient deficiencies. Plant Physiol. 169, 51–60. 10.1104/pp.15.00708 26175512 PMC4577413

[B25] Giots F. Donaton M. C. Thevelein J. M. (2003). Inorganic phosphate is sensed by specific phosphate carriers and acts in concert with glucose as a nutrient signal for activation of the protein kinase A pathway in the yeast *Saccharomyces cerevisiae*. Mol. Microbiol. 47, 1163–1181. 10.1046/j.1365-2958.2003.03365.x 12581367

[B26] Glassop D. Smith S. E. Smith F. W. (2005). Cereal phosphate transporters associated with the mycorrhizal pathway of phosphate uptake into roots. Planta 222, 688–698. 10.1007/s00425-005-0015-0 16133217

[B27] Gomez-Roldan V. Fermas S. Brewer P. B. Puech-Pagès V. Dun E. A. Pillot J.-P.. (2008). Strigolactone inhibition of shoot branching. Nature 455:189. 10.1038/nature07271 18690209

[B28] Ham B. K. Chen J. Yan Y. Lucas W. J. (2018). Insights into plant phosphate sensing and signaling. Curr. Opin. Biotechnol. 49, 1–9. 10.1016/j.copbio.2017.07.005 28732264

[B29] Hamburger D. Rezzonico E. Petétot J. M. C. Somerville C. Poirier Y. (2002). Identification and characterization of the Arabidopsis PHO1 gene involved in phosphate loading to the xylem. Plant Cell 14, 889–902. 10.1105/tpc.000745 11971143 PMC150690

[B30] Hermans C. Hammond J. P. White P. J. Verbruggen N. (2006). How do plants respond to nutrient shortage by biomass allocation? Trends Plant Sci. 11, 610–617. 10.1016/j.tplants.2006.10.007 17092760

[B31] Hoagland D. R. Arnon D. I. (1950). The Water-Culture Method for Growing Plants Without Soil. Circular. California Agricultural Experiment Station, 347.

[B32] Iyer M. K. Niknafs Y. S. Malik R. Singhal U. Sahu A. Hosono Y.. (2015). The landscape of long noncoding RNAs in the human transcriptome. Nat. Genet. 47, 199–208. 10.1038/ng.3192 25599403 PMC4417758

[B33] Jabnoune M. Secco D. Lecampion C. Robaglia C. Shu Q. Poirier Y. (2013). A rice cis-natural antisense RNA acts as a translational enhancer for its cognate mRNA and contributes to phosphate homeostasis and plant fitness. Plant Cell 25, 4166–4182. 10.1105/tpc.113.116251 24096344 PMC3877805

[B34] Jez J. M. Lee S. G. Sherp A. M. (2016). The next green movement: plant biology for the environment and sustainability. Science 353, 1241–1244. 10.1126/science.aag1698 27634525

[B35] Jiang L. Liu Y. Sun H. Han Y. Li J. Li C.. (2013). The mitochondrial folylpolyglutamate synthetase gene is required for nitrogen utilization during early seedling development in Arabidopsis. Plant Physiol. 161, 971–989. 10.1104/pp.112.203430 23129207 PMC3561033

[B36] Jung J.-Y. Ried M. K. Hothorn M. Poirier Y. (2018). Control of plant phosphate homeostasis by inositol pyrophosphates and the SPX domain. Curr. Opin. Biotechnol. 49, 156–162. 10.1016/j.copbio.2017.08.012 28889038

[B37] Kanehisa M. Araki M. Goto S. Hattori M. Hirakawa M. Itoh M.. (2007). KEGG for linking genomes to life and the environment. Nucleic Acids Res. 36, D480–D484. 10.1093/nar/gkm882 18077471 PMC2238879

[B38] Keller B. Krattinger S. G. (2017). Plant science: Genomic compartments in barley. Nature 544, 424–425. 10.1038/544424a 28447630

[B39] Khan G. A. Bouraine S. Wege S. Li Y. De Carbonnel M. Berthomieu P.. (2014). Coordination between zinc and phosphate homeostasis involves the transcription factor PHR1, the phosphate exporter PHO1, and its homologue PHO1; H3 in Arabidopsis. J. Exp. Bot. 65, 871–884. 10.1093/jxb/ert444 24420568 PMC3924728

[B40] Kochian L. V. (2012). Plant nutrition: rooting for more phosphorus. Nature 488, 466–467. 10.1038/488466a 22914160

[B41] Kong L. Zhang Y. Ye Z. Q. Liu X. Q. Zhao S. Q. Wei L.. (2007). CPC: assess the protein-coding potential of transcripts using sequence features and support vector machine. Nucleic Acids Res. 35, W345–W349. 10.1093/nar/gkm391 17631615 PMC1933232

[B42] Lan P. Li W. Schmidt W. (2012). Complementary proteome and transcriptome profiling in phosphate-deficient Arabidopsis roots reveals multiple levels of gene regulation. Mol. Cell. Proteomics 11, 1156–1166. 10.1074/mcp.M112.020461 22843991 PMC3494196

[B43] Lan P. Li W. Schmidt W. (2015). Omics approaches towards understanding plant phosphorus acquisition and use. Ann. Plant Rev. 48, 65–97. 10.1002/9781118958841.ch3

[B44] Lan T. Renner T. Ibarra-Laclette E. Farr K. M. Chang T.-H. Cervantes-Pérez S. A. (2017). Long-read sequencing uncovers the adaptive topography of a carnivorous plant genome. Proc. Natl. Acad. Sci. 14, E4435–E4441. 10.1073/pnas.1702072114 PMC5465930 28507139

[B45] Langmead B. Salzberg S. L. (2012). Fast gapped-read alignment with Bowtie 2. Nat. Methods 9:357. 10.1038/nmeth.1923 22388286 PMC3322381

[B46] Lapis-Gaza H. R. Jost R. Finnegan P. M. (2014). Arabidopsis PHOSPHATE TRANSPORTER1 genes PHT1; 8 and PHT1; 9 are involved in root-to-shoot translocation of orthophosphate. BMC Plant Biol. 14:334. 10.1186/s12870-014-0334-z 25428623 PMC4252992

[B47] Leggewie G. Willmitzer L. Riesmeier J. W. (1997). Two cDNAs from potato are able to complement a phosphate uptake-deficient yeast mutant: identification of phosphate transporters from higher plants. Plant Cell 9, 381–392. 10.1105/tpc.9.3.381 9090882 PMC156925

[B48] Li Y. Gu M. Zhang X. Zhang J. Fan H. Li P.. (2014). Engineering a sensitive visual-tracking reporter system for real-time monitoring phosphorus deficiency in tobacco. Plant Biotechnol. J. 12, 674–684. 10.1111/pbi.12171 25187932

[B49] Li Y. Niu S. Yu G. (2016). Aggravated phosphorus limitation on biomass production under increasing nitrogen loading: a meta-analysis. Glob. Chang. Biol. 22, 934–943. 10.1111/gcb.13125 26463578

[B50] Li Z. Xu C. Li K. Yan S. Qu X. Zhang J. (2012). Phosphate starvation of maize inhibits lateral root formation and alters gene expression in the lateral root primordium zone. BMC Plant Biol. 12:89. 10.1186/1471-2229-12-89 22704465 PMC3463438

[B51] Liu H. Sun M. Du D. Pan H. Cheng T. Wang J.. (2015). Whole-transcriptome analysis of differentially expressed genes in the vegetative buds, floral buds and buds of *Chrysanthemum morifolium*. PLoS ONE 10:e0128009. 10.1371/journal.pone.0128009 26009891 PMC4444331

[B52] Liu T. Y. Huang T. K. Yang S. Y. Hong Y. T. Huang S. M. Wang F. N.. (2016). Identification of plant vacuolar transporters mediating phosphate storage. Nat. Commun. 7:11095. 10.1038/ncomms11095 27029856 PMC4821872

[B53] Mascher M. Gundlach H. Himmelbach A. Beier S. Twardziok S. O. Wicker T.. (2017). A chromosome conformation capture ordered sequence of the barley genome. Nature 544, 427–433. 10.1038/nature22043 28447635

[B54] Minoche A. E. Dohm J. C. Schneider J. Holtgräwe D. Viehöver P. Montfort M.. (2015). Exploiting single-molecule transcript sequencing for eukaryotic gene prediction. Genome Biol. 16:184. 10.1186/s13059-015-0729-7 26328666 PMC4556409

[B55] Muchhal U. S. Pardo J. M. Raghothama K. (1996). Phosphate transporters from the higher plant *Arabidopsis thaliana*. Proc. Natl. Acad. Sci. U.S.A. 93, 10519–10523. 10.1073/pnas.93.19.10519 8927627 PMC38418

[B56] Muehe E. M. Eisele J. F. Daus B. Kappler A. Harter K. Chaban C. (2014). Are rice (*Oryza sativa* L.) phosphate transporters regulated similarly by phosphate and arsenate? a comprehensive study. Plant Mol. Biol. 85, 301–316. 10.1007/s11103-014-0186-9 24729002

[B57] Murphy J. Riley J. P. (1962). A modified single solution method for the determination of phosphate in natural waters. Anal. Chim. Acta 27, 31–36. 10.1016/S0003-2670(00)88444-5

[B58] Nussaume L. Kanno S. Javot H. Marin E. Pochon N. Ayadi A.. (2011). Phosphate import in plants: focus on the PHT1 transporters. Front. Plant Sci. 2:83. 10.3389/fpls.2011.00083 22645553 PMC3355772

[B59] Okazaki Y. Otsuki H. Narisawa T. Kobayashi M. Sawai S. Kamide Y.. (2013). A new class of plant lipid is essential for protection against phosphorus depletion. Nat. Commun. 4:1510. 10.1038/ncomms2512 23443538 PMC3586718

[B60] Oono Y. Kawahara Y. Yazawa T. Kanamori H. Kuramata M. Yamagata H.. (2013). Diversity in the complexity of phosphate starvation transcriptomes among rice cultivars based on RNA-Seq profiles. Plant Mol. Biol. 83, 523–537. 10.1007/s11103-013-0106-4 23857470 PMC3830200

[B61] Popova Y. Thayumanavan P. Lonati E. Agrochão M. Thevelein J. M. (2010). Transport and signaling through the phosphate-binding site of the yeast Pho84 phosphate transceptor. Proc. Natl. Acad. Sci. 107, 2890–2895. 10.1073/pnas.0906546107 20133652 PMC2840322

[B62] Pourkheirandish M. Hensel G. Kilian B. Senthil N. Chen G. Sameri M.. (2015). Evolution of the grain dispersal system in barley. Cell 162, 527–539. 10.1016/j.cell.2015.07.002 26232223

[B63] Preuss C. P. Huang C. Y. Gilliham M. Tyerman S. D. (2010). Channel-like characteristics of the low-affinity barley phosphate transporter PHT1; 6 when expressed in *Xenopus oocytes*. Plant Physiol. 152, 1431–1441. 10.1104/pp.109.152009 20053709 PMC2832247

[B64] Punta M. Coggill P. C. Eberhardt R. Y. Mistry J. Tate J. Boursnell C.. (2011). The Pfam protein families database. Nucleic Acids Res. 40, D290–D301. 10.1093/nar/gkr1065 22127870 PMC3245129

[B65] Quan X. Zeng J. Ye L. Chen G. Han Z. Shah J. M.. (2016). Transcriptome profiling analysis for two Tibetan wild barley genotypes in responses to low nitrogen. BMC Plant Biol. 16:30. 10.1186/s12870-016-0721-8 26817455 PMC4728812

[B66] Rae A. L. Cybinski D. H. Jarmey J. M. Smith F. W. (2003). Characterization of two phosphate transporters from barley; evidence for diverse function and kinetic properties among members of the Pht1 family. Plant Mol. Biol. 53, 27–36. 10.1023/B:PLAN.0000009259.75314.15 14756304

[B67] Raven J. A. (2015). Interactions between nitrogen and phosphorus metabolism. Annu. Plant Rev. 48, 187–214. 10.1002/9781118958841.ch7

[B68] Ren P. Ma X. Li B. Meng Y. Lai Y. Si E. (2016). Identification and selection of low-phosphate-tolerant germplasm in barley (*Hordeum vulgare* L.). Soil Sci. Plant Nutr. 62, 471–480. 10.1080/00380768.2016.1223521

[B69] Royo B. Moran J. F. Ratcliffe R. G. Gupta K. J. (2015). Nitric oxide induces the alternative oxidase pathway in Arabidopsis seedlings deprived of inorganic phosphate. J. Exp. Bot. 66, 6273–6280. 10.1093/jxb/erv338 26163703 PMC4588884

[B70] Ruyter-Spira C. Kohlen W. Charnikhova T. Van Zeijl A. Van Bezouwen L. Cardoso C.. (2011). Physiological effects of the synthetic strigolactone analog GR24 on root system architecture in Arabidopsis: another belowground role for strigolactones? Plant Physiol. 155, 721–734. 10.1104/pp.110.166645 21119044 PMC3032462

[B71] Sage R. F. Mckown A. D. (2005). Is C4 photosynthesis less phenotypically plastic than C3 photosynthesis? J. Exp. Bot. 57, 303–317. 10.1093/jxb/erj040 16364950

[B72] Schünmann P. Richardson A. Smith F. Delhaize E. (2004). Characterization of promoter expression patterns derived from the Pht1 phosphate transporter genes of barley (*Hordeum vulgare* L.). J. Exp. Bot. 55, 855–865. 10.1093/jxb/erh103 15020637

[B73] Secco D. Jabnoune M. Walker H. Shou H. Wu P. Poirier Y.. (2013). Spatio-temporal transcript profiling of rice roots and shoots in response to phosphate starvation and recovery. Plant Cell 25, 4285–4304. 10.1105/tpc.113.117325 24249833 PMC3875719

[B74] Shemi A. Schatz D. Fredricks H. F. Van Mooy B. A. Porat Z. Vardi A.. (2016). Phosphorus starvation induces membrane remodeling and recycling in Emiliania huxleyi. New Phytol. 211, 886–898. 10.1111/nph.13940 27111716

[B75] Shin H. Shin H. S. Dewbre G. R. Harrison M. J. (2004). Phosphate transport in Arabidopsis: Pht1; 1 and Pht1; 4 play a major role in phosphate acquisition from both low- and high- phosphate environments. Plant J. 39, 629–642. 10.1111/j.1365-313X.2004.02161.x 15272879

[B76] Siebers M. Dörmann P. Hölzl G. (2015). Membrane remodelling in phosphorus-deficient plants. Annu. Plant Rev. 48, 237–263. 10.1002/9781118958841.ch9

[B77] Smith F. W. Cybinski D. H. Rae A. L. (1999). Regulation of expression of genes encoding phosphate transporters in barley roots, in Plant Nutrition-Molecular Biology and Genetics, eds Gissel-Nielsen G. Jensen A. (Springer), 145–150.

[B78] Steijger T. Abril J. F. Engström P. G. Kokocinski F. Akerman M. Guigó R.. (2013). Assessment of transcript reconstruction methods for RNA-seq. Nat. Methods 10:1177. 10.1038/nmeth.2714 24185837 PMC3851240

[B79] Tian Y. Feng F. Zhang B. Li M. Wang F. Gu L.. (2017). Transcriptome analysis reveals metabolic alteration due to consecutive monoculture and abiotic stress stimuli in *Rehamannia glutinosa* Libosch. Plant Cell Rep. 36, 859–875. 10.1007/s00299-017-2115-2 28275853

[B80] Tilgner H. Jahanbani F. Blauwkamp T. Moshrefi A. Jaeger E. Chen F.. (2015). Comprehensive transcriptome analysis using synthetic long-read sequencing reveals molecular co-association of distant splicing events. Nat. Biotechnol. 33, 736–742. 10.1038/nbt.3242 25985263 PMC4832928

[B81] Tong Y. Zhang W. Wang X. Couture R. M. Larssen T. Li J. (2017). Decline in Chinese lake phosphorus concentration accompanied by shift in sources since 2006. Nat. Geosci. 10, 507–511. 10.1038/ngeo2967

[B82] Trapnell C. Pachter L. Salzberg S. L. (2009). TopHat: discovering splice junctions with RNA-Seq. Bioinformatics 25, 1105–1111. 10.1093/bioinformatics/btp120 19289445 PMC2672628

[B83] Trapnell C. Roberts A. Goff L. Pertea G. Kim D. Kelley D. R.. (2012). Differential gene and transcript expression analysis of RNA-seq experiments with TopHat and Cufflinks. Nat. Protoc. 7:562. 10.1038/nprot.2012.016 22383036 PMC3334321

[B84] Umehara M. Hanada A. Magome H. Takeda-Kamiya N. Yamaguchi S. (2010). Contribution of strigolactones to the inhibition of tiller bud outgrowth under phosphate deficiency in rice. Plant Cell Physiol. 51, 1118–1126. 10.1093/pcp/pcq084 20542891 PMC2900824

[B85] Van Mooy B. Krupke A. Dyhrman S. T. Fredricks H. F. Frischkorn K. R. Ossolinski J. E. (2015). Major role of planktonic phosphate reduction in the marine phosphorus redox cycle. Science 348, 783–785. 10.1126/science.aaa8181 25977548

[B86] VanBuren R. Bryant D. Edger P. P. Tang H. Burgess D. Challabathula D.. (2015). Single-molecule sequencing of the desiccation-tolerant grass Oropetium thomaeum. Nature 527:508. 10.1038/nature15714 26560029

[B87] Vergutz L. Manzoni S. Porporato A. Novais R. F. Jackson R. B. (2012). Global resorption efficiencies and concentrations of carbon and nutrients in leaves of terrestrial plants. Ecol. Monogr. 82, 205–220. 10.1890/11-0416.1

[B88] Wang B. Tseng E. Regulski M. Clark T. A. Hon T. Jiao Y.. (2016). Unveiling the complexity of the maize transcriptome by single-molecule long-read sequencing. Nat. Commun. 7:11708. 10.1038/ncomms11708 27339440 PMC4931018

[B89] Wang H. Xu Q. Kong Y. H. Chen Y. Duan J. Y. Chen Y. F.. (2014). Arabidopsis WRKY45 transcription factor activates PHOSPHATE TRANSPORTER1;1 expression in response to phosphate starvation. Plant Physiol. 164, 2020–2029. 10.1104/pp.113.235077 24586044 PMC3982759

[B90] Wang Z. Ruan W. Shi J. Zhang L. Xiang D. Yang C.. (2014). Rice SPX1 and SPX2 inhibit phosphate starvation responses through interacting with PHR2 in a phosphate-dependent manner. Proc. Natl. Acad. Sci. U.S.A. 111, 14953–14958. 10.1073/pnas.1404680111 25271318 PMC4205599

[B91] Wu P. Shou H. Xu G. Lian X. (2013). Improvement of phosphorus efficiency in rice on the basis of understanding phosphate signaling and homeostasis. Curr. Opin. Plant Biol. 16, 205–212. 10.1016/j.pbi.2013.03.002 23566853

[B92] Xu Z. Peters R. J. Weirather J. Luo H. Liao B. Zhang X.. (2015). Full-length transcriptome sequences and splice variants obtained by a combination of sequencing platforms applied to different root tissues of Salvia miltiorrhiza and tanshinone biosynthesis. Plant J. 82, 951–961. 10.1111/tpj.12865 25912611

[B93] Yamaji N. Takemoto Y. Miyaji T. Mitani-Ueno N. Yoshida K. T. Ma J. F.. (2017). Reducing phosphorus accumulation in rice grains with an impaired transporter in the node. Nature 541:92. 10.1038/nature20610 28002408

[B94] Yan J. Yu L. Xuan J. Lu Y. Lu S. Zhua W.. (2016). De novo transcriptome sequencing and gene expression profiling of spinach (*Spinacia oleracea* L.) leaves under heat stress. Sci. Rep. 6:19473. 10.1038/srep19473 26857466 PMC4746569

[B95] Young M. D. Wakefield M. J. Smyth G. K. Oshlack A. (2010). Gene ontology analysis for RNA-seq: accounting for selection bias. Genome Biol. 11:R14. 10.1186/gb-2010-11-2-r14 20132535 PMC2872874

[B96] Zeng J. He X. Wu D. Zhu B. Cai S. Nadira U. A.. (2014). Comparative transcriptome profiling of two Tibetan wild barley genotypes in responses to low potassium. PLoS ONE 9:e100567. 10.1371/journal.pone.0100567 24949953 PMC4065039

[B97] Zhang Z. Liao H. Lucas W. J. (2014). Molecular mechanisms underlying phosphate sensing, signaling, and adaptation in plants. J. Integr. Plant Biol. 56, 192–220. 10.1111/jipb.12163 24417933

[B98] Zhu X. F. Zhu C. Q. Wang C. Dong X. Y. Shen R. F. (2017). Nitric oxide acts upstream of ethylene in cell wall phosphorus reutilization in phosphorus-deficient rice. J. Exp. Bot. 68, 753–760. 10.1093/jxb/erw480 28064177 PMC6055659

