# Supplementary material for: Molecular Mechanisms of Acclimatization to Phosphorus Starvation and Recovery Underlying Full-Length Transcriptome Profiling in Barley (Hordeum vulgare L.)
Source: Front Plant Sci. 2018 Apr 18;9:500. doi: 10.3389/fpls.2018.00500 (PMC5915550; doi:10.3389/fpls.2018.00500)
Supplement: Supplemental Figure 1 — Annular appearance of data comparison between the barley genome and PacBio sequencing. Different colors express different numbers (number ≤ 10 color = deep red, number < 20 color = red, number < 30 color = light red, number < 40 color = deep yellow, number < 50 color = yellow, number < 60 color = light yellow, number < 70 color = light green, number < 80 color = green, number < 90 color = deep green, number < 100 color = light blue, number < 110 color = blue, number < 120 color = deep blue, number >= 120 color = black). (A) Chromosomes of the barley genome. (B) Gene distribution of the genome and PacBio sequencing. (C) Transcript distribution of the genome and PacBio sequencing. (D) Allocation of alternative splicing events on chromosomes. (E) Allocation of long non-coding RNAs on chromosomes. (F) Allocation of fusion genes on chromosomes. Black lines represent intra-chromosome fusion genes. Orange lines represent inter-chromosome fusion genes. [file Presentation1.pdf]

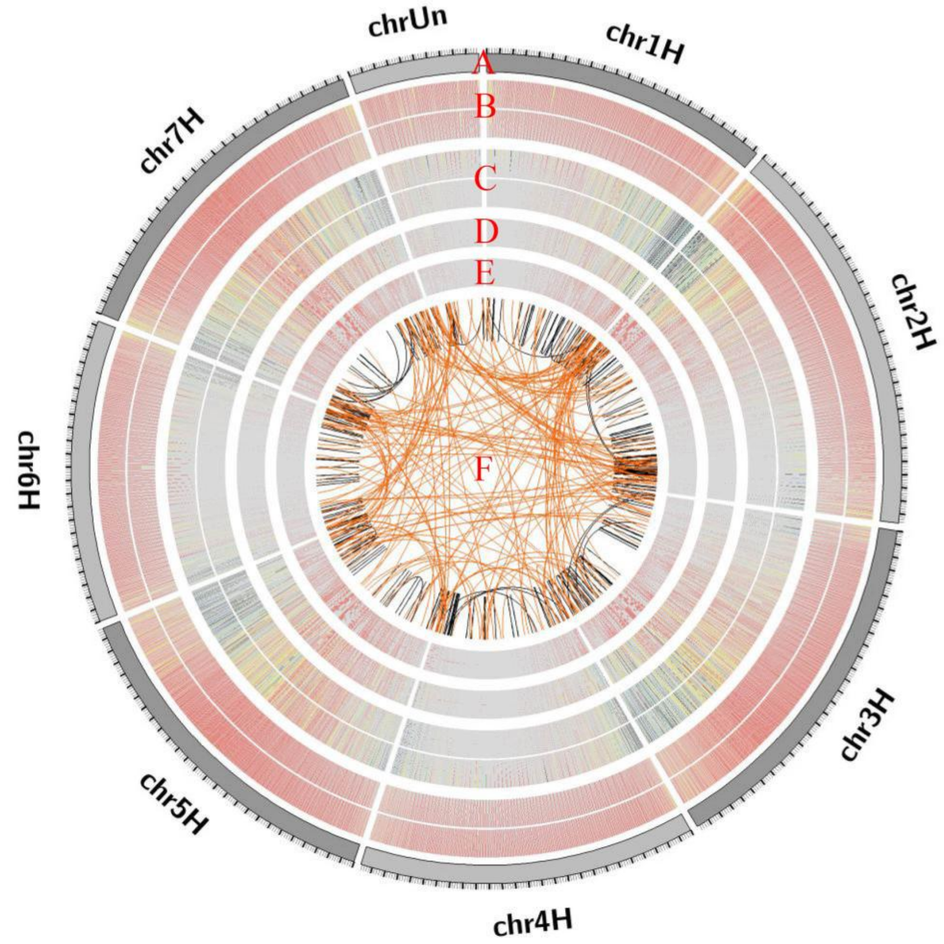

Supplemental Figure 1. Annular appearance of data comparison between the barley genome and PacBio sequencing. Different colors express different numbers (number <= 10 color = deep red, number < 20 color = red, number < 30 color= light red, number < 40 color = deep yellow, number < 50 color = yellow, number < 60 color = light yellow, number < 70 color = light green, number < 80 color = green, number < 90 color = deep green, number < 100 color = light blue, number < 110 color = blue, number < 120 color = deep blue, number >= 120 color = black). (A) Chromosomes of the barley genome. (B) Gene distribution of the genome and PacBio sequencing. (C) Transcript distribution of the genome and PacBio sequencing. (D) Allocation of alternative splicing events on chromosomes. (E) Allocation of long non-coding RNAs on chromosomes. (F) Allocation of fusion genes on chromosomes. Black lines represent intra-chromosome fusion genes. Orange lines represent inter-chromosome fusion genes.

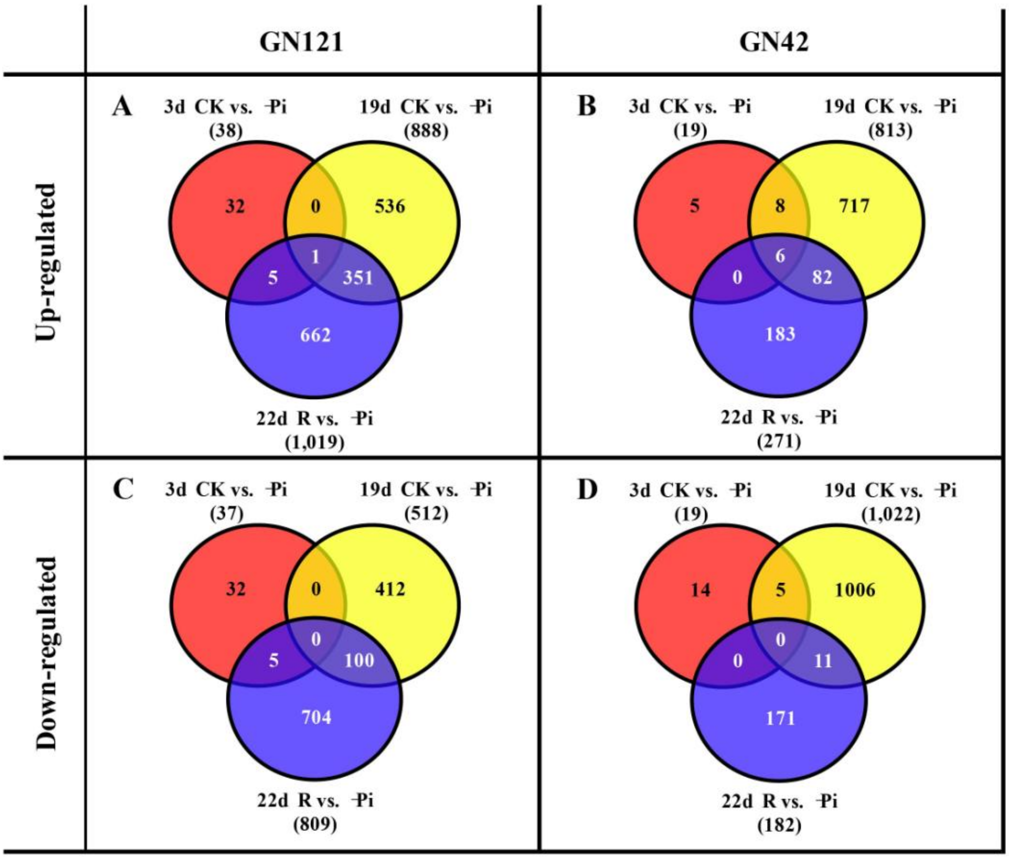

Supplemental Figure 2. Venn diagrams of differentially expressed genes (DEGs) between the treatment and control groups in the leaves of GN121 and GN42 at three time points. (A) Number of up-regulated genes in GN121. (B) Number of up-regulated genes in GN42. (C) Number of down-regulated genes in GN121. (D) Number of down -regulated genes in GN42.

Supplemental Figure 3. Quantitative reverse-transcription PCR validation of differentially expressed genes in roots and leaves. Black columns represent FPKM based on the Illumina platform. The red broken lines represent relative expression quantity tested by qRT-PCR. The bars represent SE (n = 3).

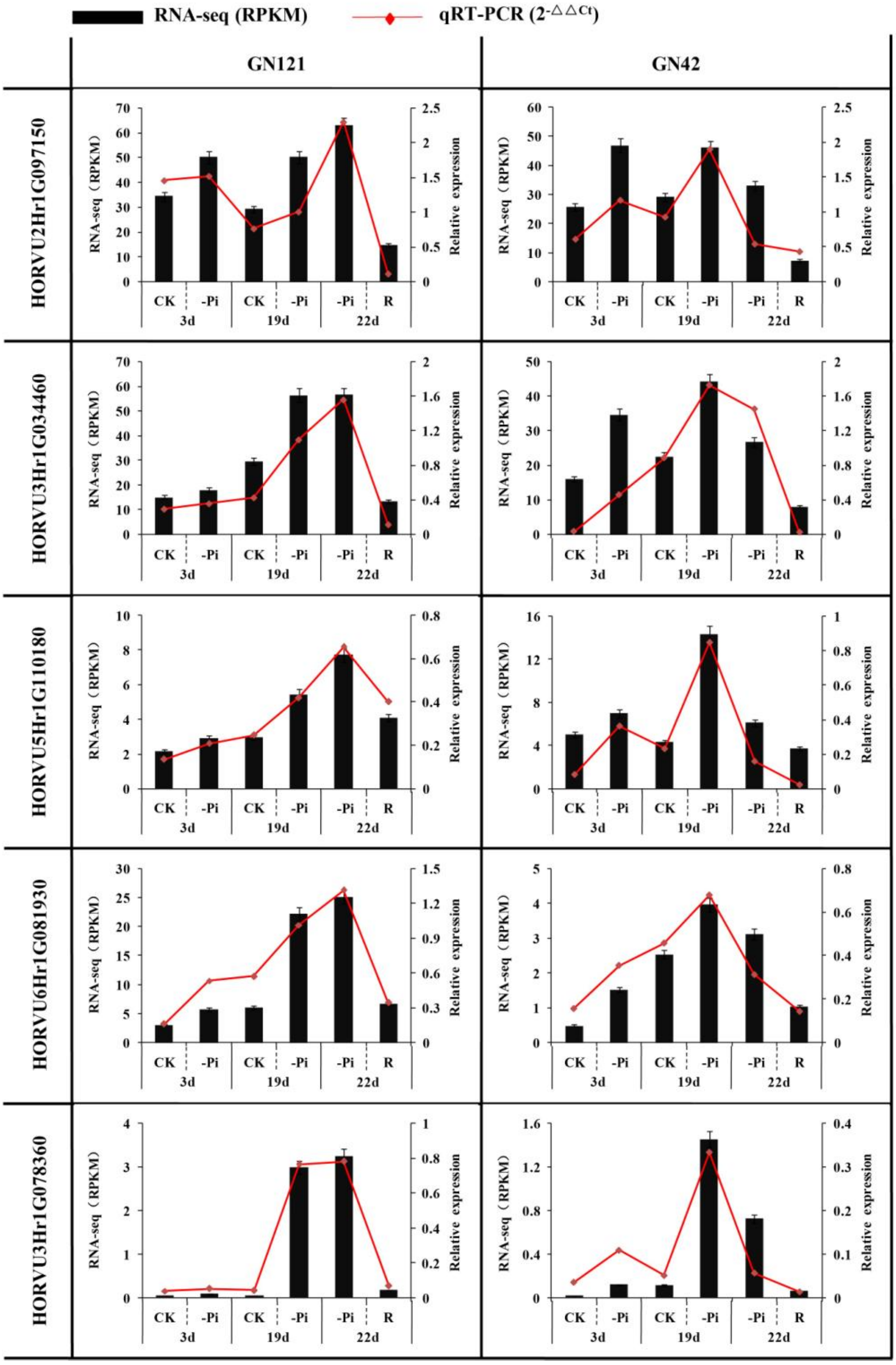

Supplemental Figure 3 (A) Expression levels of five differentially expressed genes based on RNA-seq and qRT-PCR in roots.

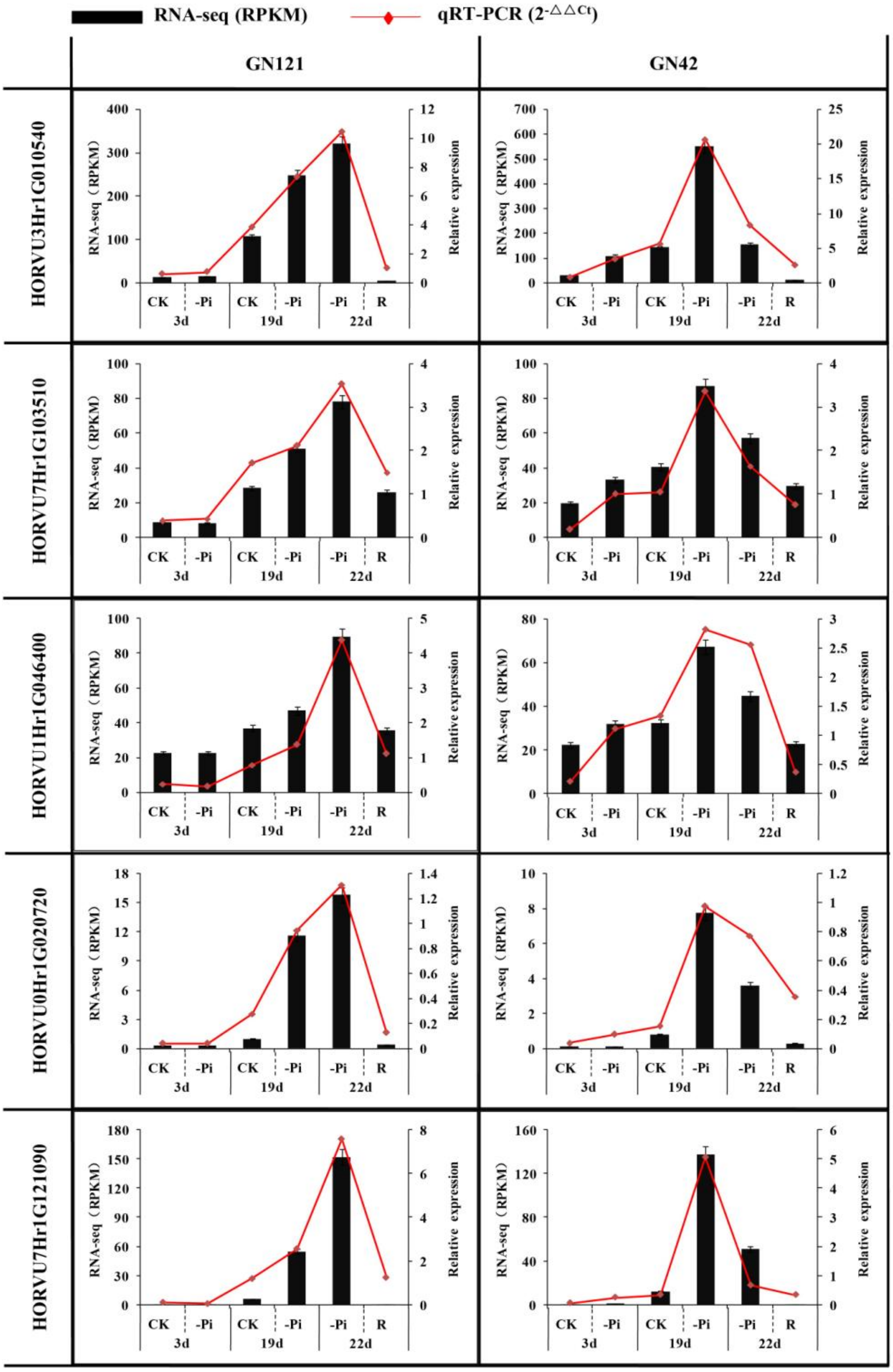

Supplemental Figure 3 (B) Expression levels of five differentially expressed genes based on RNA-seq and qRT-PCR in leaves.

Supplemental Figure 4. Gene Ontology (GO) analysis of differentially expressed genes in the roots of GN121 and GN42. Red columns represent the biological process (BP). Green columns represent the cellular component (CC). Blue columns represent the molecular function (MF).

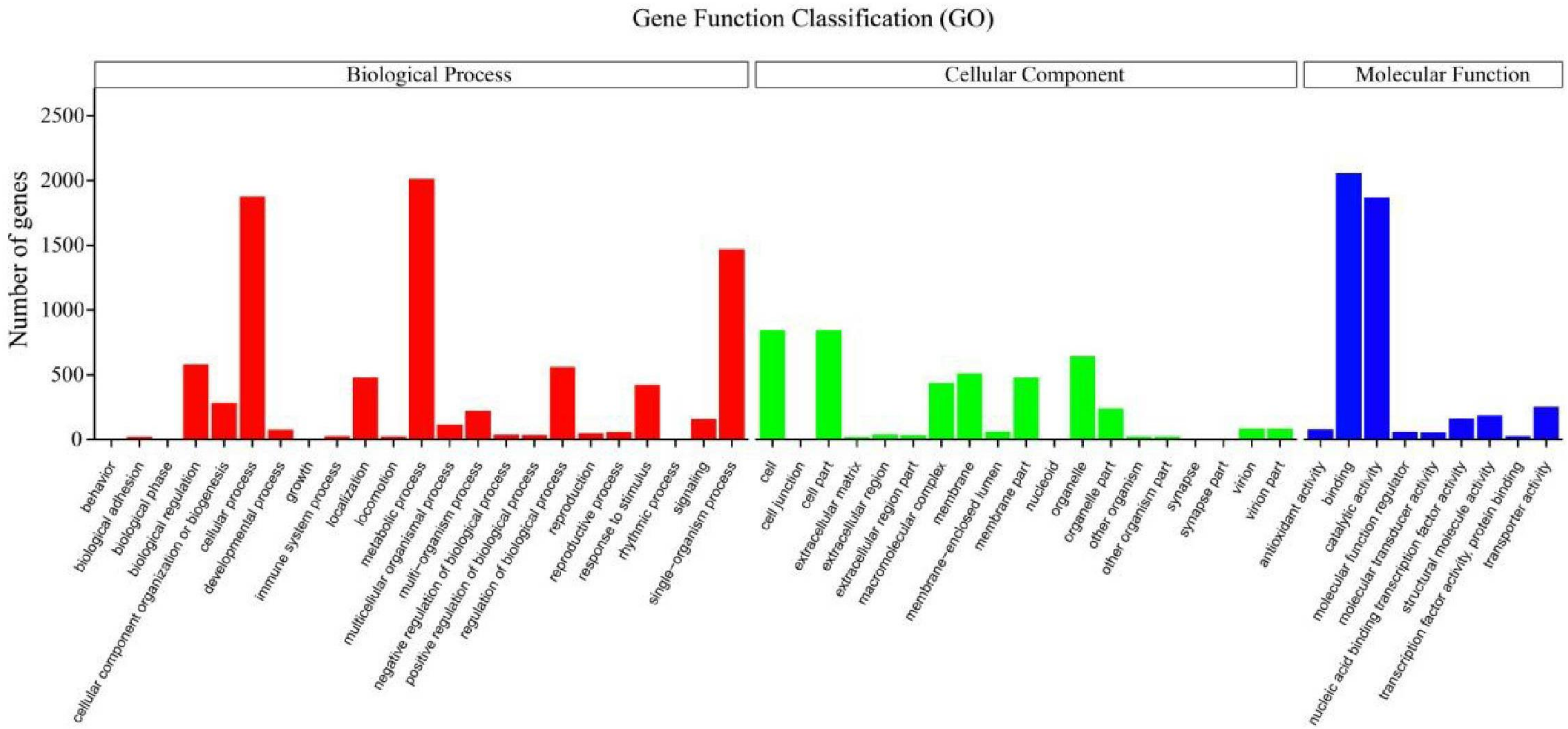

Supplemental Figure 4 (A) Gene number of GO enrichment terms in the roots of GN121.

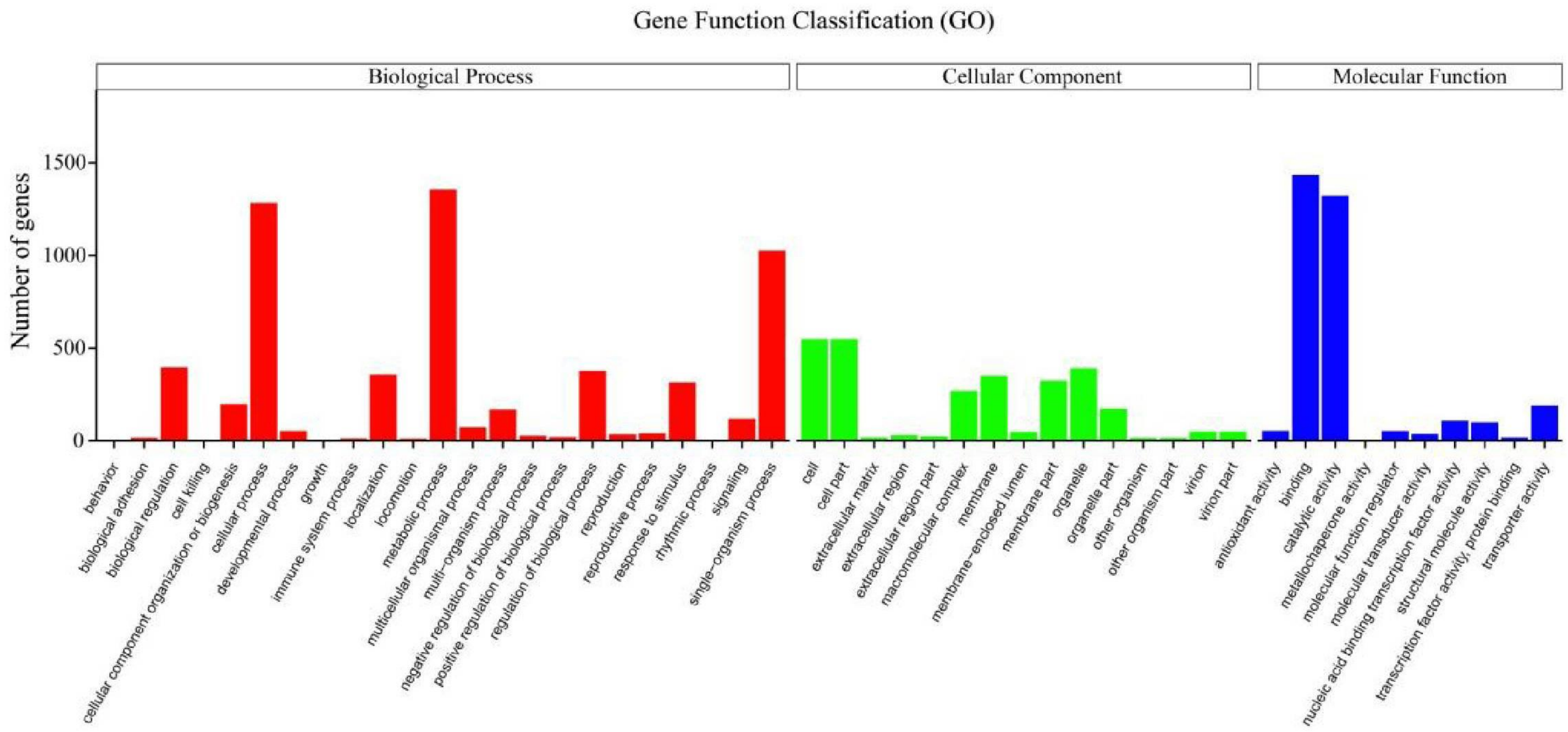

Supplemental Figure 4 (B) Gene number of GO enrichment terms in the roots of GN42.

Supplemental Figure 5. Gene Ontology (GO) analysis of differentially expressed genes in the leaves of GN121 and GN42. Red columns represent the biological process (BP). Green columns represent the cellular component (CC). Blue columns represent the molecular function (MF).

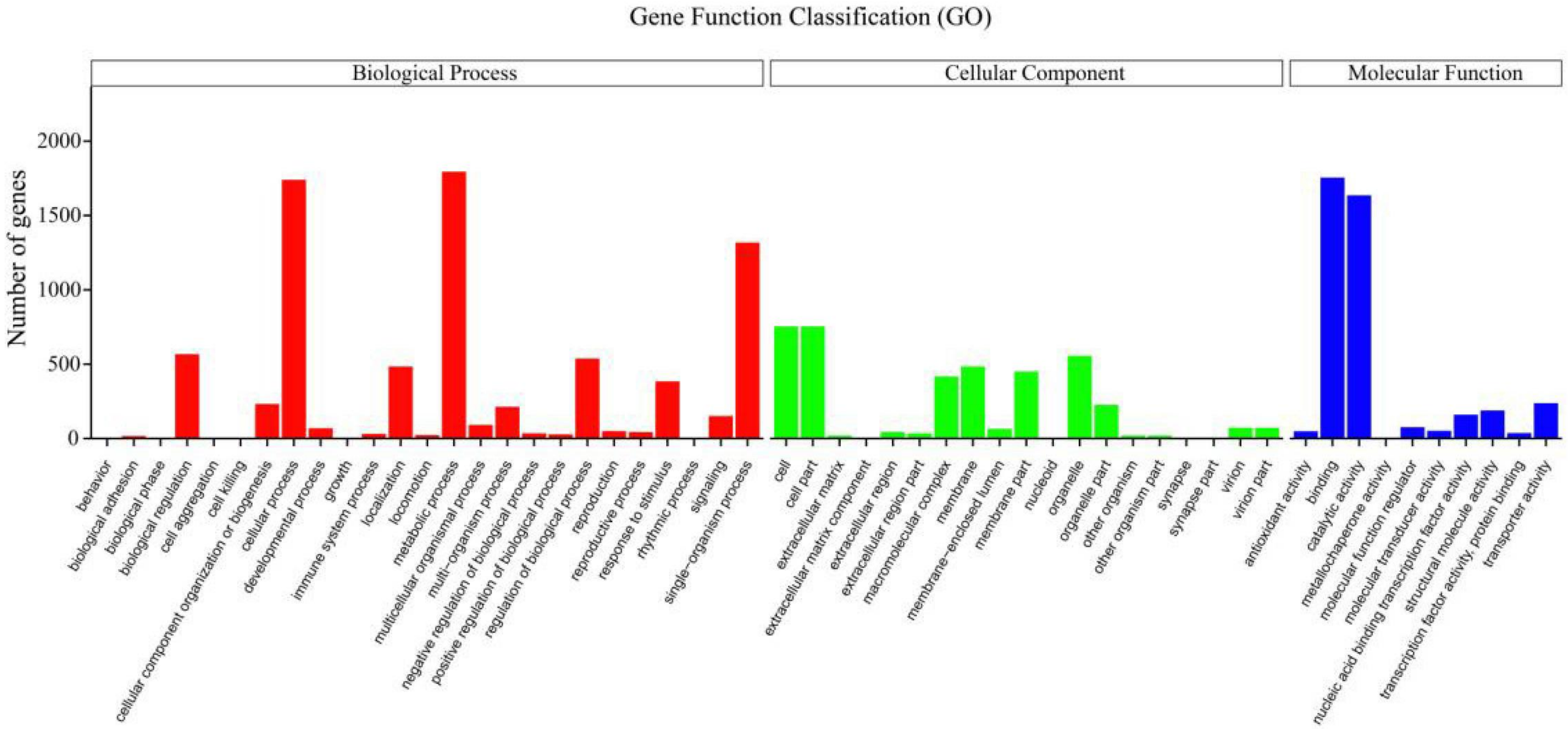

Supplemental Figure 5 (A) Gene number of GO enrichment terms in the leaves of GN121.

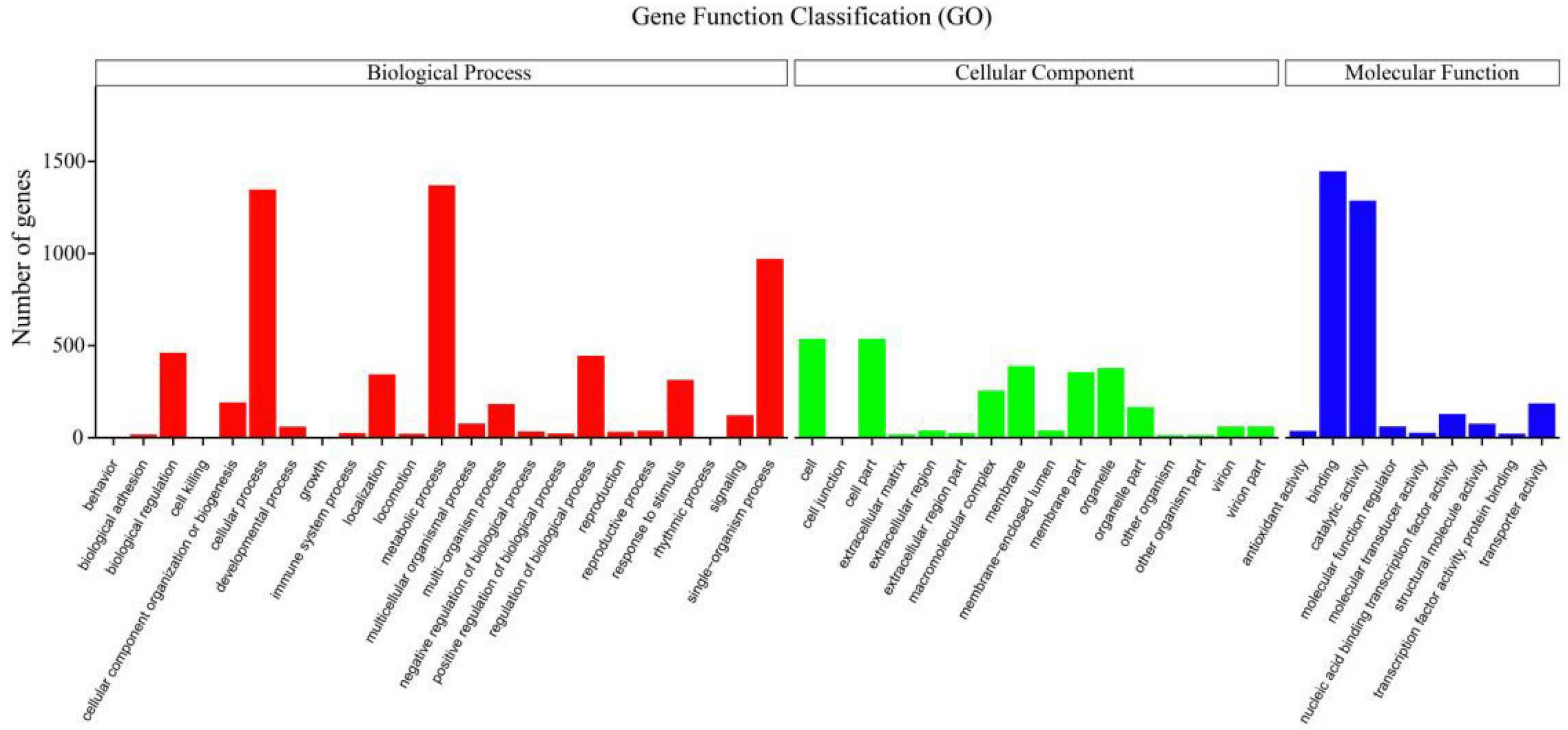

Supplemental Figure 5 (B) Gene number of GO enrichment terms in the leaves of GN42.

Supplemental Figure 6. KEGG analysis of differentially expressed genes in the roots of GN121 and GN42.

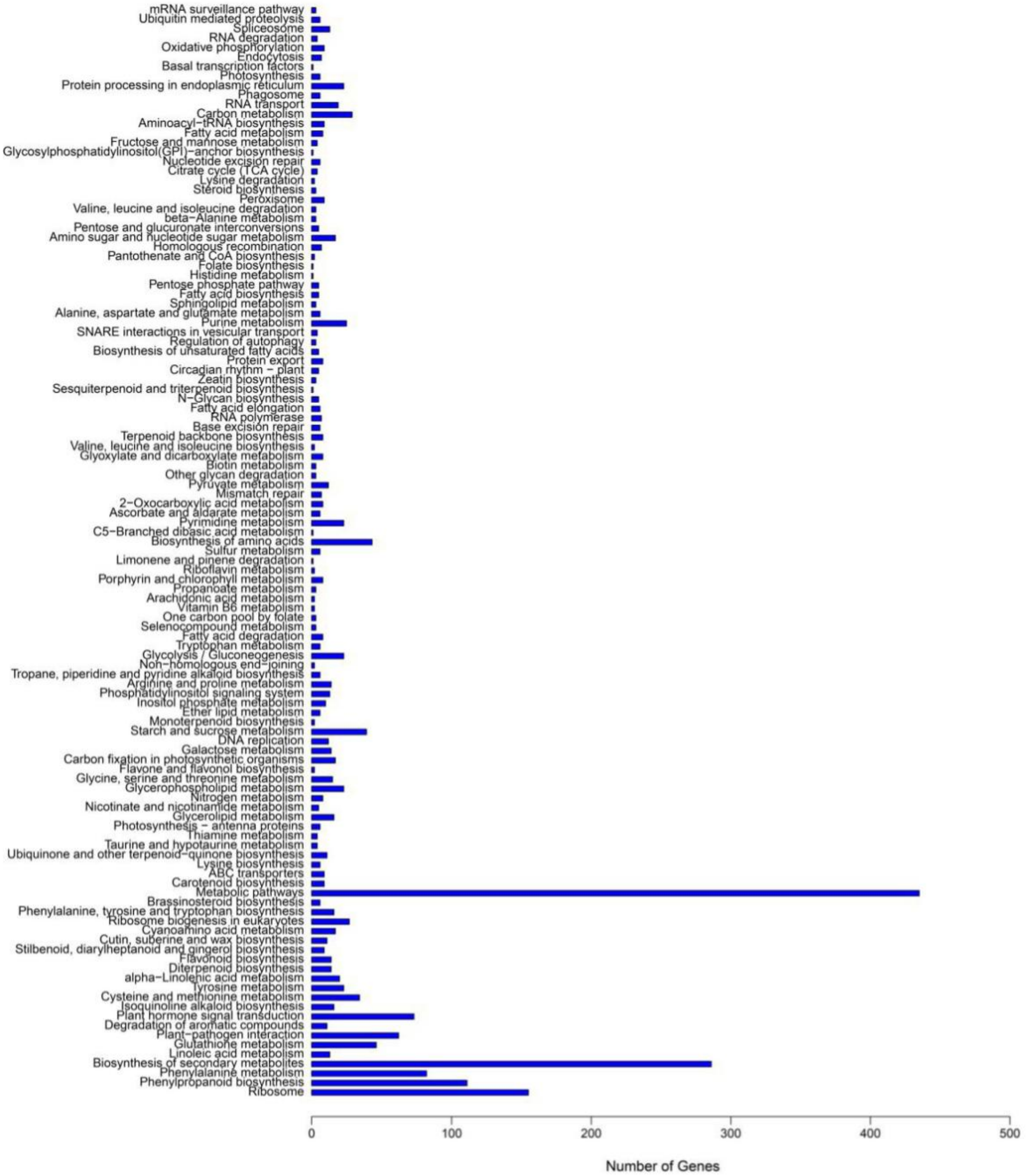

Supplemental Figure 6 (A) Gene number of KEGG enrichment terms in the roots of GN121.

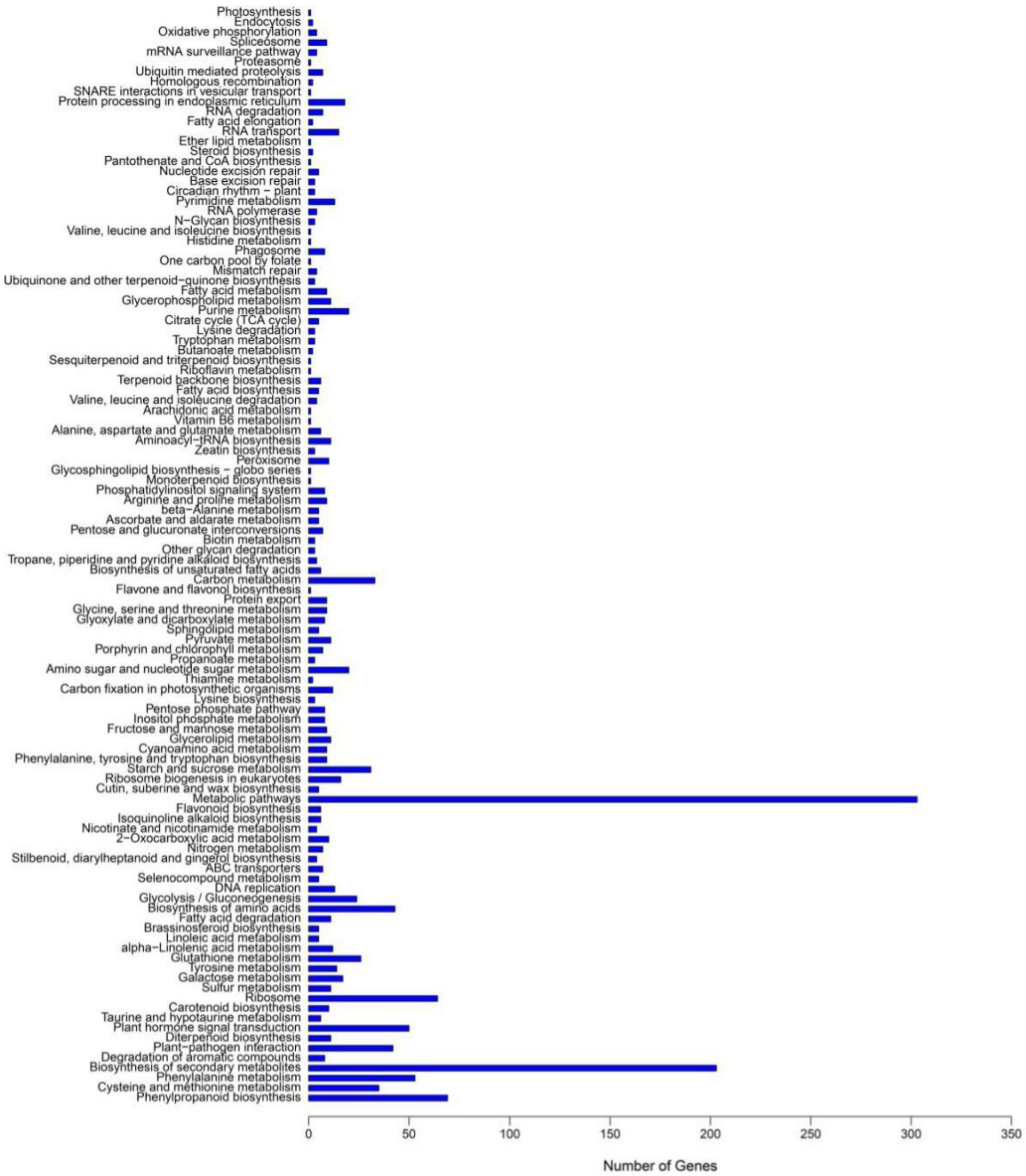

Supplemental Figure 6 (B) Gene number of KEGG enrichment terms in the roots of GN42.

Supplemental Figure 7. KEGG analysis of differentially expressed genes in the leaves of GN121 and GN42.

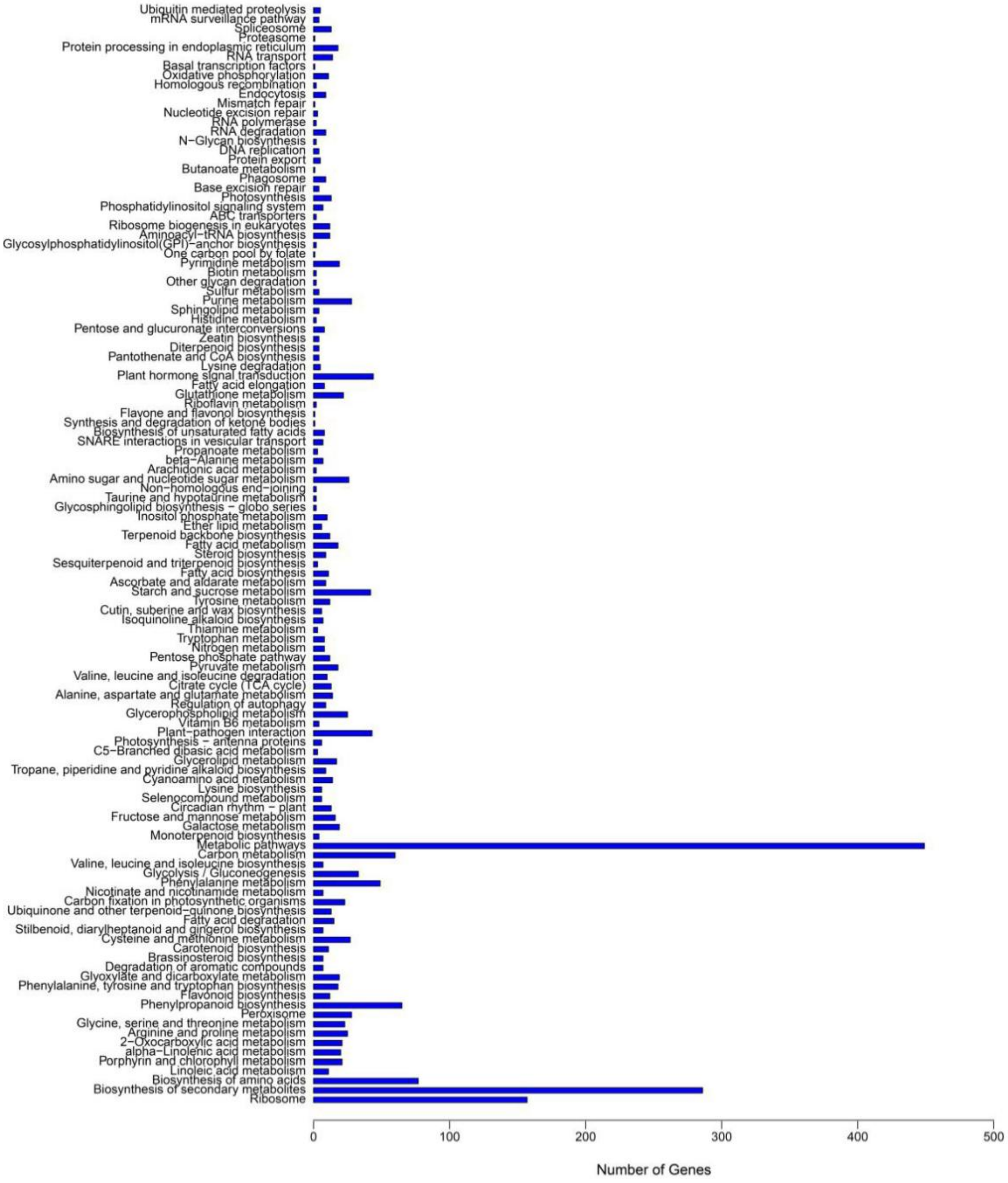

Supplemental Figure 7 (A) Gene number of KEGG enrichment terms in the leaves of GN121.

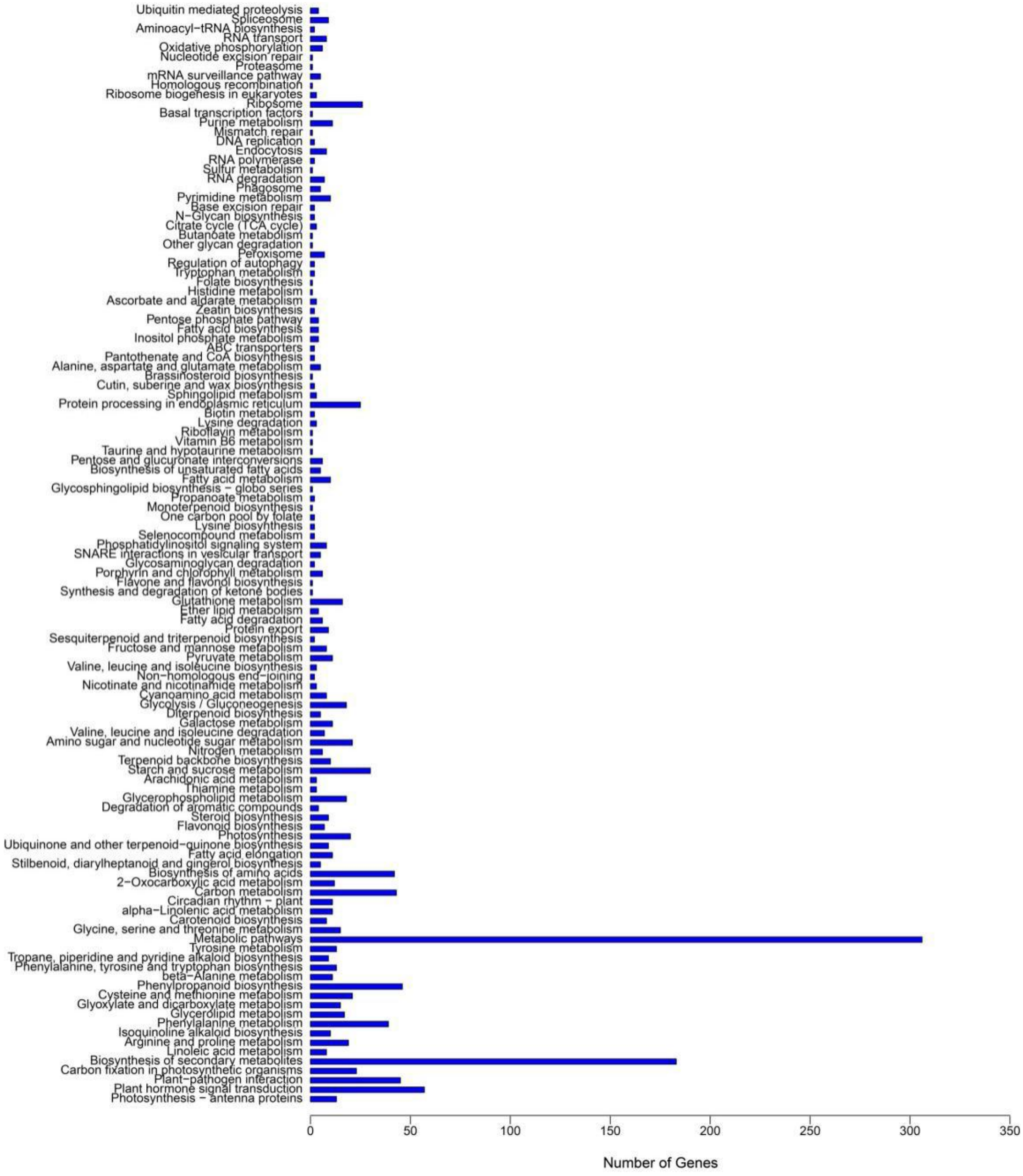

Supplemental Figure 7 (B) Gene number of KEGG enrichment terms in the leaves of GN42.

Supplemental Figure 8. Heat maps of key differentially expressed genes involved in the phosphorus metabolic pathway at three time points in roots and leaves (CK/ R, normal phosphorus level. –Pi, low phosphorus level).

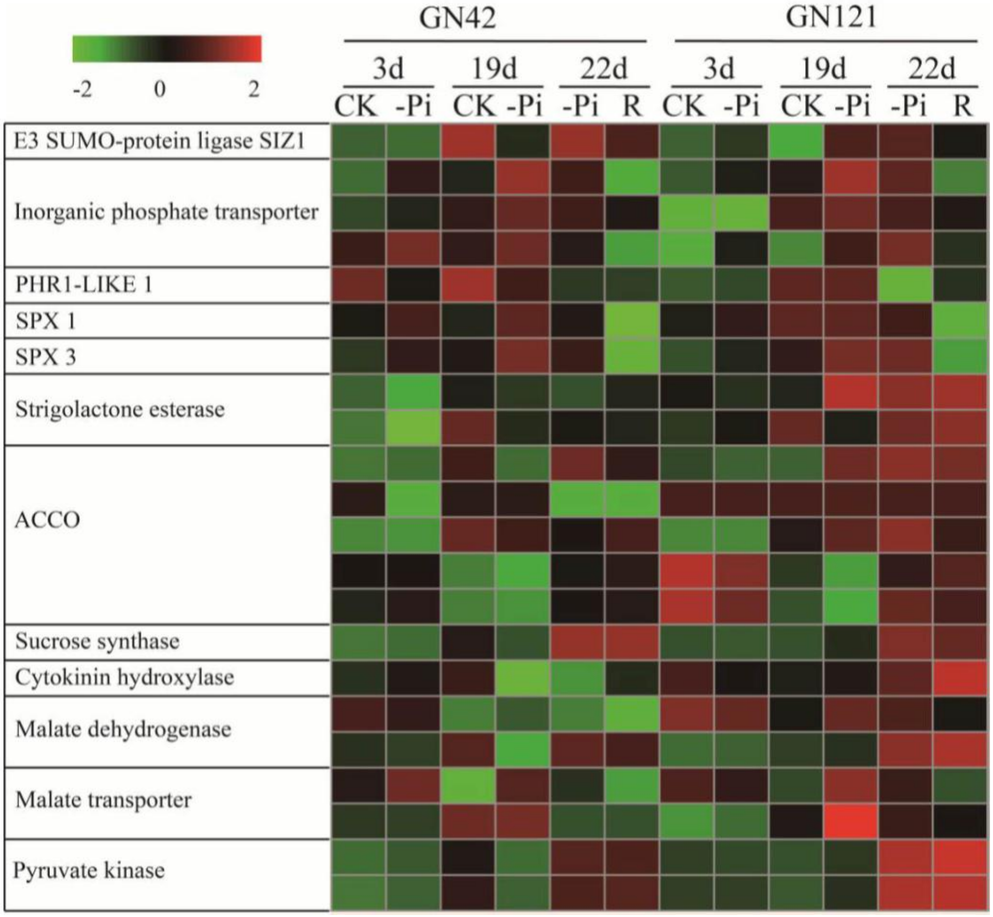

Supplemental Figure 8 (A) Heat map of DEGs that participated in phosphorus metabolism of roots.

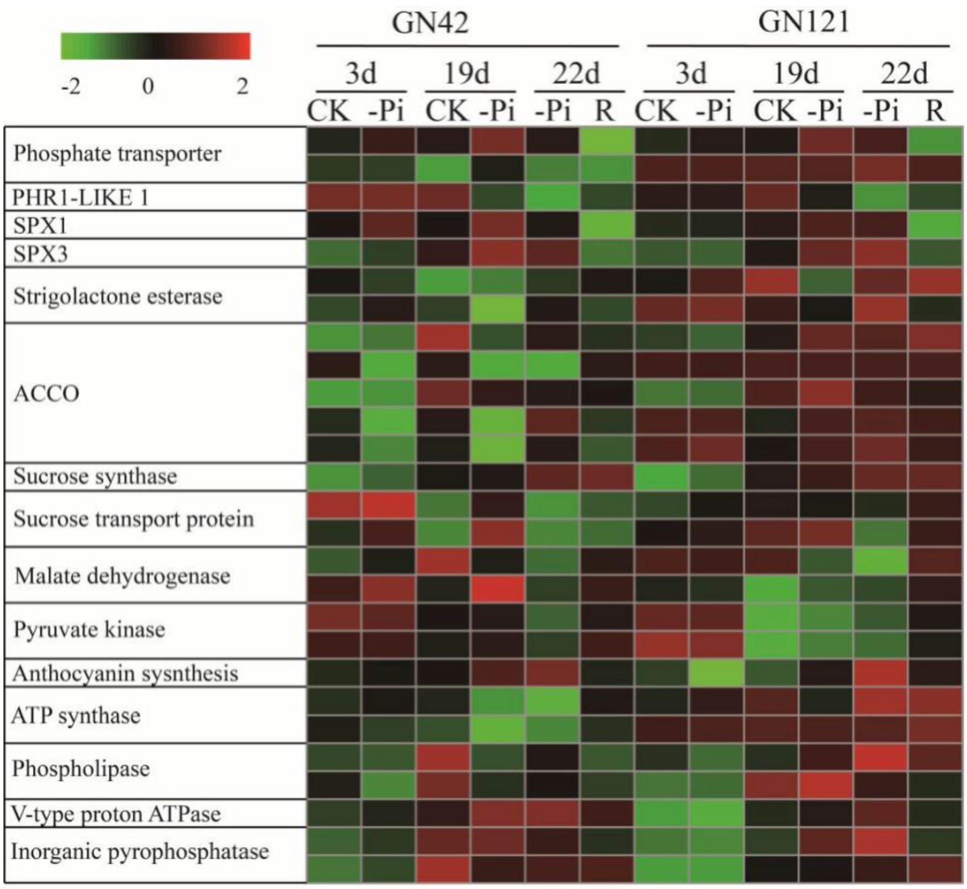

Supplemental Figure 8 (B) Heat map of DEGs that participated in phosphorus metabolism of leaves.
