# Supplementary material for: Molecular Mechanisms of Acclimatization to Phosphorus Starvation and Recovery Underlying Full-Length Transcriptome Profiling in Barley (Hordeum vulgare L.)
Source: Front Plant Sci. 2018 Apr 18;9:500. doi: 10.3389/fpls.2018.00500 (PMC5915550; doi:10.3389/fpls.2018.00500)
Supplement: Supplemental Table 8 — List of the co-expressed differentially expressed genes between GN121 and GN42 in roots and leaves. List of the 32 co-expressed differentially expressed genes (DEGs) between GN121 and GN42 at three time points in roots and leaves (–Pi 19d, low phosphorus treatment for 19 d. –Pi 22d, low phosphorus treatment for 22 d.). [file Table8.DOC]

| **Serial number** | **Gene ID** | **GN42 (FPKM)** | | | **GN121 (FPKM)** | | | **Description** |
| --- | --- | --- | --- | --- | --- | --- | --- | --- |
|  |  | **-Pi 19d** | **-Pi 22d** | **Fold Change** | **-Pi 19d** | **-Pi 22d** | **Fold Change** |  |
| **Shoot** | | | | | | | | |
| 1 | HORVU4Hr1G079600 | 1005.65 | 1006.75 | 1.00 | 1262.95 | 1546.46 | 1.22 | -//- |
| 2 | HORVU2Hr1G031400 | 378.50 | 76.58 | 0.20 | 132.67 | 332.94 | 2.51 | SPX domain-containing protein 5 |
| 3 | HORVU5Hr1G117630 | 99.95 | 52.22 | 0.52 | 12.00 | 21.21 | 1.77 | Uncharacterized protein |
| 4 | HORVU3Hr1G034460 | 37.58 | 17.82 | 0.47 | 30.56 | 40.17 | 1.31 | Purple acid phosphatase 15 |
| 5 | HORVU7Hr1G089910 | 80.42 | 31.03 | 0.39 | 53.42 | 51.33 | 0.96 | SPX domain-containing protein 1 |
| 6 | HORVU3Hr1G068380 | 246.39 | 59.35 | 0.24 | 143.89 | 209.66 | 1.46 | Inorganic pyrophosphatase 2 |
| 7 | HORVU5Hr1G005290 | 1700.27 | 1818.13 | 1.07 | 1566.87 | 2289.64 | 1.46 | Thaumatin-like protein |
| **Root** | | | | | | | | |
| 1 | HORVU2Hr1G064140 | 21.53 | 10.06 | 0.47 | 21.84 | 12.99 | 0.59 | Probable disease resistance protein |
| 2 | HORVU2Hr1G020140 | 110.55 | 41.15 | 0.37 | 93.83 | 60.52 | 0.64 | Transcription factor BOA |
| 3 | HORVU3Hr1G076060 | 133.35 | 91.10 | 0.68 | 149.38 | 112.50 | 0.75 | Purple acid phosphatase 2 |
| 4 | HORVU7Hr1G117400 | 35.02 | 31.92 | 0.91 | 26.16 | 33.45 | 1.28 | U-box domain-containing protein 33 |
| 5 | HORVU5Hr1G062450 | 53.58 | 20.94 | 0.39 | 21.25 | 28.92 | 1.36 | -//- |
| 6 | HORVU3Hr1G076320 | 90.76 | 48.64 | 0.54 | 95.87 | 36.33 | 0.38 | Inositol hexakisphosphate  and diphosphoinositol-pentakisphosphate kinase 1 |
| 7 | HORVU0Hr1G020720 | 42.16 | 21.96 | 0.52 | 43.82 | 28.01 | 0.64 | Probable inorganic phosphate transporter 1-10 |
| 8 | HORVU3Hr1G013420 | 9.87 | 4.37 | 0.44 | 9.78 | 8.41 | 0.86 | Cytochrome P450 |
| 9 | HORVU3Hr1G079900 | 667.37 | 320.07 | 0.48 | 700.58 | 444.60 | 0.63 | Glycerophosphodiester phosphodiesterase GDPD1 |
| 10 | HORVU3Hr1G010540 | 204.18 | 36.66 | 0.18 | 149.62 | 49.97 | 0.33 | Sulfoquinovosyl transferase SQD2 |
| 11 | HORVU4Hr1G044140 | 102.35 | 71.92 | 0.70 | 129.35 | 87.52 | 0.68 | Probable monogalactosyldiacylglycerol synthase 2 |
| 12 | HORVU7Hr1G113020 | 78.38 | 18.92 | 0.24 | 106.95 | 41.34 | 0.39 | membrane protein |
| 13 | HORVU2Hr1G013130 | 61.31 | 23.30 | 0.38 | 41.88 | 27.95 | 0.67 | U-box domain-containing protein 33 |
| 14 | HORVU3Hr1G013490 | 132.96 | 63.89 | 0.48 | 114.95 | 61.64 | 0.54 | Cytochrome P450 |
| 15 | HORVU7Hr1G071290 | 53.42 | 31.37 | 0.59 | 50.57 | 46.46 | 0.92 | Putative glycerol-3-phosphate transporter 1 |
| 16 | HORVU5Hr1G094880 | 4.13 | 1.81 | 0.44 | 2.42 | 1.82 | 0.75 | Probable receptor-like protein kinase |
| 17 | HORVU3Hr1G013430 | 4.83 | 2.18 | 0.45 | 5.09 | 3.59 | 0.71 | 5-epiaristolochene 1,3-dihydroxylase |
| 18 | HORVU3Hr1G091170 | 24.79 | 11.69 | 0.47 | 37.76 | 22.72 | 0.60 | Serine/threonine-protein kinase CDG1 |
| 19 | HORVU6Hr1G005930 | 70.55 | 21.02 | 0.30 | 24.94 | 20.22 | 0.81 | High-affinity nitrate transporter 2.2 |
| 20 | HORVU7Hr1G121090 | 151.22 | 51.90 | 0.34 | 151.73 | 148.27 | 0.98 | SPX domain-containing protein 3 |
| 21 | HORVU5Hr1G055570 | 16.17 | 10.67 | 0.66 | 20.21 | 19.44 | 0.96 | Purple acid phosphatase 15 |
| 22 | HORVU7Hr1G083970 | 65.52 | 32.35 | 0.49 | 80.22 | 35.99 | 0.45 | NADP-dependent alkenal double bond reductase P2 |
| 23 | HORVU2Hr1G031400 | 450.31 | 135.85 | 0.30 | 400.36 | 227.85 | 0.57 | SPX domain-containing protein 5 |
| 24 | HORVU3Hr1G068380 | 223.29 | 50.66 | 0.23 | 199.81 | 117.63 | 0.59 | Inorganic pyrophosphatase 2 |
| 25 | HORVU7Hr1G089910 | 117.95 | 54.08 | 0.46 | 117.47 | 81.60 | 0.69 | SPX domain-containing protein 1 |
