# Supplementary material for: Molecular Mechanisms of Acclimatization to Phosphorus Starvation and Recovery Underlying Full-Length Transcriptome Profiling in Barley (Hordeum vulgare L.)
Source: Front Plant Sci. 2018 Apr 18;9:500. doi: 10.3389/fpls.2018.00500 (PMC5915550; doi:10.3389/fpls.2018.00500)
Supplement: Supplemental Table 9 — List of differentially expressed genes related to phosphorus metabolism in leaves. List of DEGs related to phosphorus metabolism in leaves (–Pi, treated with low phosphorus solution. CK/Pi recovery, treated with normal phosphorus solution). [file Table9.DOC]

| **Gene ID** | **GN121 Log2(Fold Changes)** | | | **GN42 Log2(Fold Changes)** | | | **Annotation** |
| --- | --- | --- | --- | --- | --- | --- | --- |
|  | **3d -Pi/CK** | **19d -Pi/CK** | **22d -Pi/R** | **3d -Pi/CK** | **19d -Pi/CK** | **22d -Pi/R** |  |
| **Transporter** | |  |  |  |  |  |  |
| HORVU4Hr1G083550 |  |  | 1.718 |  |  |  | Inorganic phosphate transporter 1-2 |
| HORVU5Hr1G110180 |  |  | 1.9851 |  |  |  | Inorganic phosphate transporter 1-4 |
| HORVU5Hr1G110220 |  |  | 1.6077 |  |  |  | Inorganic phosphate transporter 1-4 |
| HORVU4Hr1G080730 |  |  | ∞ |  |  |  | Inorganic phosphate transporter 1-7 |
| HORVU5Hr1G117080 |  |  | 5.0087 |  | 1.3983 |  | Inorganic phosphate transporter 1-8 |
| HORVU0Hr1G020720 |  | 3.6777 | 5.483 |  | 3.2713 | 3.6819 | Inorganic phosphate transporter 1-10 |
| HORVU7Hr1G047210 |  | 1.5763 | 2.9724 |  | 1.4168 |  | Phosphate transporter PHO1-3 |
| **Transcription** | |  |  |  |  |  |  |
| HORVU6Hr1G065710 |  |  | 2.3312 |  | 1.6583 | 2.1902 | SPX domain-containing membrane protein |
| HORVU7Hr1G089910 |  | 0.55378 | 2.8715 | 1.0569 | 1.4226 | 2.6086 | SPX domain-containing protein 1 |
| HORVU7Hr1G121090 |  | 3.3044 | 8.5871 |  | 3.5017 | 8.0509 | SPX domain-containing protein 3 |
| HORVU2Hr1G031400 |  |  | 6.3383 | 2.4931 | 3.6322 | 5.4205 | SPX domain-containing protein 5 |
| **Lipid metabolism** | |  |  |  |  |  |  |
| HORVU2Hr1G073850 |  |  |  |  | -0.93641 |  | Digalactosyldiacylglycerol synthase 1 |
| HORVU4Hr1G020770 |  | 0.58367 | 1.4819 |  |  | 1.0266 | Digalactosyldiacylglycerol synthase 1 |
| HORVU4Hr1G067920 |  | 0.66668 | 0.69974 |  |  |  | Digalactosyldiacylglycerol synthase 2 |
| HORVU2Hr1G070700 |  | 2.2997 | 3.7494 |  |  |  | Glycerophosphodiester phosphodiesterase GDPD1 |
| HORVU3Hr1G079900 |  | 0.9976 | 5.8728 |  | 2.1257 | 5.6367 | Glycerophosphodiester phosphodiesterase GDPD1 |
| HORVU2Hr1G032670 |  |  | ∞ |  |  |  | Glycerophosphodiester phosphodiesterase GDPD6 |
| HORVU4Hr1G044140 |  | 1.3159 | 6.5814 |  | 2.1339 | 6.9951 | Monogalactosyldiacylglycerol synthase 2 |
| HORVU6Hr1G087090 |  | 1.076 | 2.123 |  | 1.0455 | 1.4764 | Monogalactosyldiacylglycerol synthase 3 |
| HORVU2Hr1G065110 |  | 0.82522 | 1.6295 |  |  |  | Sulfoquinovosyl transferase SQD2 |
| HORVU3Hr1G010540 |  | 1.28 | 6.0814 |  |  |  | Sulfoquinovosyl transferase SQD2 |
| ***continued*** |  |  |  |  |  |  |  |
| HORVU4Hr1G061040 |  |  | 0.98782 |  |  |  | Sulfoquinovosyl transferase SQD2 |
| **Metabolism** |  |  |  |  |  |  |  |
| HORVU1Hr1G054430 |  | 1.6435 | 2.6162 |  | 1.5359 | 2.2522 | Haloacid dehalogenase-like hydrolase domain-containing protein Sgpp |
| HORVU6Hr1G090040 |  | 1.0239 |  |  |  |  | Probable nicotianamine synthase 4 |
| HORVU2Hr1G050140 |  | 0.86516 | 0.82488 |  |  |  | Pyruvate kinase isozyme A |
| HORVU1Hr1G054380 |  | 1.3111 |  |  |  | 1.0452 | Pyruvate kinase isozyme G |
| **Phosphorylation/Dephosphorylation** | | |  |  |  |  |  |
| HORVU4Hr1G064360 |  | 1.7546 | 3.1615 |  |  | 1.8145 | Purple acid phosphatase 17 |
| HORVU5Hr1G042160 |  |  | 1.3151 |  |  | 0.90909 | Purple acid phosphatase 22 |
| **Miscellaneous** |  |  |  |  |  |  |  |
| HORVU5Hr1G074270 |  |  |  |  | -0.73254 |  | Chaperone protein dnaJ 10 |
| HORVU2Hr1G120010 |  |  | 1.2739 |  |  |  | Chaperone protein dnaJ 11 |
| HORVU2Hr1G103310 |  |  | 1.0567 |  |  |  | Nucleoredoxin 1-2 |
| HORVU3Hr1G009240 |  |  |  |  | -1.7789 |  | Protein FAR-RED ELONGATED HYPOCOTYL 3 |
