# Supplementary material for: Molecular Mechanisms of Acclimatization to Phosphorus Starvation and Recovery Underlying Full-Length Transcriptome Profiling in Barley (Hordeum vulgare L.)
Source: Front Plant Sci. 2018 Apr 18;9:500. doi: 10.3389/fpls.2018.00500 (PMC5915550; doi:10.3389/fpls.2018.00500)
Supplement: Supplemental Table 10 — Primers for quantitative reverse-transcription PCR. [file Table10.DOC]

| Tissue | Gene ID | Primer sequence (From 5′ to 3′) |
| --- | --- | --- |
| Root | HORVU2Hr1G097150 | F: GGGTAGCATAAAGCCGTTGG |
|  |  | A: GTGTTGTTCGCCGCACCT |
|  | HORVU3Hr1G034460 | F: GTTGGCGGCATCCATTTCA |
|  |  | A: CCTCCCTGTAGTGAGCCTTGTAG |
|  | HORVU5Hr1G110180 | F: GGCCTTCCCGTACCACC |
|  |  | A: CCGTGGTCCACCTTGCTG |
|  | HORVU6Hr1G081930 | F: AATCGGCGCATCAGAAGG |
|  |  | A: GCCATCCAGCAGGGGAC |
|  | HORVU3Hr1G078360 | F: TGGGACTTCTTCCTTCACCTCA |
|  |  | A: GATGAAGTGGACGCTCCCTT |
| Shoot | HORVU3Hr1G010540 | F: ATGTCGTCGGGTGTCCCT |
|  |  | A: CCTCCAGTCGCACTTCTCC |
|  | HORVU7Hr1G103510 | F: ATGGGAAGTTTCTGGCTTTAGG |
|  |  | A: GTCAGCGGGCACATTTAGTTTT |
|  | HORVU1Hr1G046400 | F: TACGACTGCAATGTCCCTCTTCA |
|  |  | A: GGAACTGCTAGGAATGGCTTGA |
|  | HORVU0Hr1G020720 | F: GTCCGCGACCATCATGTCC |
|  |  | A: TAAACCTGGCTGTTTCTGGCA |
|  | HORVU7Hr1G121090 | F: AAGGTGAGGAGGGAGATCGTG |
|  |  | A: GGACGAGCCTGGACATGAGC |
| *HvActin* (AY145451) | | F: GCCGTGCTTTCCCTCTATG |
|  |  | A: GCTTCTCCTTGATGTCCCTTA |
